# Analgesic efficacy of adding the IPACK block to multimodal analgesia protocol for primary total knee arthroplasty: a meta-analysis of randomized controlled trials

**DOI:** 10.1186/s13018-022-03266-3

**Published:** 2022-09-29

**Authors:** Xiumei Tang, Yahao Lai, Siwei Du, Ning Ning

**Affiliations:** 1 West China School of Nursing, Sichuan University, #37 Guoxue Road, Chengdu, 610041 People’s Republic of China; 2 Department of Orthopedics, West China Hospital, Sichuan University, #37 Guoxue Road, Chengdu, 610041 People’s Republic of China; 3 Department of Orthopedics, Orthopedic Research Institute, West China Hospital, Sichuan University, Chengdu, 610041 People’s Republic of China

**Keywords:** IPACK block, Total knee arthroplasty, Randomized controlled trial, Meta-analysis

## Abstract

**Background:**

Total knee arthroplasty (TKA) is a standard treatment for end-stage degenerative knee disease. Most patients will experience moderate-to-severe postoperative knee pain, significantly affecting rehabilitation. However, controversy remains regarding the efficacy of adding the interspace between the popliteal artery and capsule of the knee (IPACK) into multimodal analgesia protocol.

**Methods:**

PubMed, Medline, Embase, Cochrane Library, and other databases were searched from inception to February 1, 2021. Studies comparing patients receiving IPACK to patients not receiving IPACK were included. The primary outcome was the ambulation pain score on a visual analogue scale (VAS) of 0–10. Secondary outcomes included pain score at rest, morphine usage, functional recovery, clinical outcomes, and complications.

**Results:**

Thirteen RCTs involving 1347 knees were included. IPACK was associated with lower ambulation pain scores (weight mean difference [WMD] − 0.49, 95% confidence interval [CI] − 0.72 to − 0.26). The benefits were observed from 2 to 4 h, 6 to 12 h, and beyond one week. IPACK also significantly reduced rest pain scores (WMD − 0.49, 95% CI − 0.74 to − 0.24), and the benefits were observed from 6 to 12 h and beyond one week. IPACK reduced the overall morphine consumption (WMD − 2.56, 95% CI − 4.63 to − 0.49). Subgroup analysis found reduced oral morphine consumption from 24 to 48 h (WMD − 2.98, 95% CI − 5.71 to − 0.24) and reduced rate of morphine requirement from 12 to 24 h (relative risk [RR] = 0.51, 95% CI 0.31 to 0.83). Functional recovery outcomes regarding ambulation distances (on the second postoperative day [POD2]) (WMD = 1.74, 95% CI 0.34 to 3.15) and quadriceps muscle strength (at 0 degree) (WMD = 0.41, 95% CI 0.04 to 0.77) favored IPACK. And IPACK reduced the rate of sleep disturbance (on POD 1) (RR = 0.39, 95% CI 0.19 to 0.81). There was no significant difference in the other outcomes.

**Conclusions:**

Moderate-level evidence confirmed that IPACK was related to better results in pain scores, morphine usage, and functional recovery without increasing the risk of complications.

***Registration*:**

CRD42021252156.

**Supplementary Information:**

The online version contains supplementary material available at 10.1186/s13018-022-03266-3.

## Key points

- **Question** Can IPACK as an additional analgesic method provide better results on postoperative knee pain and function recovery for patients after TKA?
- **Findings** Moderate evidence suggested that the addition of IPACK to themultimodal analgesia programs had better results on postoperative knee pain VAS scores, morphine usage, ambulation distances, muscle strength, and sleep disturbance without increasing the risk of complications, compared to those without IPACK. However, these differences in pain VAS scores, ambulation distances, and muscle strength were minor and had relative clinical significance. The reduced morphine consumption significantly confirmed the benefits of IPACK.
- **Meaning** The combinations of IPACK with other regional analgesia techniques (e.i. PAI, ACB) are recommended as an integral part of multimodal analgesia programs. More trials were needed to confirm the benefits of IPACK in different combinations.

**Supplementary Information:**

The online version contains supplementary material available at 10.1186/s13018-022-03266-3.

## Background

Total knee arthroplasty (TKA) is an effective intervention for end-stage knee diseases and could relieve pain, restore function, and improve patients’ quality of life [1]. However, patients usually experience moderate-to-severe postoperative knee pain [2]. Due to osteophytes removal and soft tissue release on the backside of the knee, posterior knee pain is also a significant issue [3]. Insufficient pain control may hinder early ambulation, hamper the quality of recovery, and increase the utilization of opioids [4].

The interspace between the popliteal artery and capsule of the knee (IPACK) is a novel regional anesthetic approach that could supply analgesic effects on the posterior capsule without compromising muscle strength [5]. Cadaveric data demonstrated that IPACK mainly anesthetizes the articular branches from the tibial and obturator nerves [6]. Several randomized controlled trials (RCTs) reported the benefits of IPACK complemented many regional anesthesia modalities [3, 7–12]. However, these studies yielded conflicting results regarding the use of IPACK for analgesia after TKA. Three studies [7, 10, 13] reported lower pain visual analogue scale (VAS) scores, while the other two studies [3, 14] found similar pain scores with the addition of IPACK. Two studies [12, 15] found longer postoperative ambulation distances in the IPACK group, while the other three studies had contract results [3, 11, 16]. IPACK has been adopted into clinical practice, but the efficacy of IPACK has not been confirmed by synthesized evidence. Two reviews discussed the efficacy of IPACK in the practice of multimodal pain management. However, their conclusions lacked the support of quantity information, and the certainty of evidence cannot be measured. Moreover, previous studies found that the analgesic effect of IPACK usually disappeared within 24 h, while the long-term effects were unclear.

Therefore, we conducted a systematic review and meta-analysis to ascertain the benefit of IPACK in combination with other analgesic methods concerning (1) pain scores (at rest, at ambulation); (2) morphine consumption (amount and frequency); (3) functional recovery (range of motion, muscle strength, ambulation distances, time-up-and-go test time); (4) complications (needle puncture, postoperative nausea, vomiting, sleep disturbance); and (5) clinical outcomes (length of stay, operation duration, patients satisfaction).

## Methods

This review was reported according to the criteria of the Preferred Reporting Items for Systematic Reviews and Meta-Analyses (PRISMA) statement (Additional file 1) [17]. The protocol for this review was registered with the International Prospective Register of Systematic Reviews (PROSPERO—CRD42021252156).

### Search strategy

We searched for databases including PubMed, Medline, Embase, the Cochrane Library, Ovid, Web of Science, and websites including Clinicaltrials.gov, WHO International Clinical Trials Registry Platform (ICTRP), and Google Scholar till February 1, 2021. The following terms were used: (IPACK OR “interspace between the popliteal artery and posterior capsule of the knee”) AND (total knee arthroplasty OR knee arthroplasty OR total knee replacement OR knee replacement OR TKA OR TKR) AND ((randomize* control* trial*) OR RCT)). No language or date limits were placed on the search. We also used a manual search strategy, checked references, and contacted authors to identify additional studies. Two authors screened studies with a third author adjudicating in case of disagreement.

### Trial selection

The studies had to be RCTs comparing TKA patients with IPACK. Any non-RCTs, quasi-RCTs, retrospective studies, cadaver studies, comments, letters, editorials, protocols, guidelines, surgical registries, and review papers were excluded. Follow-up reports at different time points or different comparisons in one trial will be extracted separately. Studies with multiple arms were eligible, as were studies in which multiple regional anesthetic techniques were performed, so long as an IPACK was one of the arms or one of the used techniques. There was no restriction on language or publishing year. Two investigators independently screened titles and abstracts to exclude non-relevant trials. Discrepancies were resolved by a third author. Relevant full-text articles were retrieved and analyzed for eligibility using the pre-defined inclusion criteria.

### Data extraction

Data were extracted via a standardized spreadsheet according to a pre-agreed protocol. The following information was collected: first author, publication year, country, number of participants in each group, patient demographics, inclusion and exclusion criteria, and conclusions. We collected: interventions, dosages, and types of anesthesia drug administered, the method of anesthesia, pain rescue methods, multimodal analgesia protocol, surgeons, prothesis, approach, follow-up duration, and numbers of patients lost to follow. If data cannot be extracted directly or missing, we will contact the authors by email or calculate data with the Cochrane Review Manager calculator [18]. Two authors independently extracted the information, and any discrepancies were resolved by a third author. Pain scores reported on visual, verbal, or numerical rating scales were converted to a standardized 0–10 scale. All opioids were converted to oral milligram morphine equivalents via an online website (http://opioidcalculator.practicalpainmanagement.com/).

### Outcomes

The primary outcome was the ambulation pain score. The secondary outcomes were rest pain score, morphine consumption, functional recovery outcomes, clinical outcomes, and complications. The morphine consumption was collected as a continuous variable (amount) and category variable (used or not). The functional recovery outcomes included the range of motion (ROM), quadriceps muscle strength (QMS), ambulation distances, and time-up-and-go test (TUG) time. The clinical outcomes included the length of hospital stay, operation time, and patient satisfaction. The complications were postoperative nausea and vomiting (PONV) and sleep disturbance.

### Subgroup analyses

Our pre-defined subgroup analysis was based on multiple time points. The subgroups were as closest to 6, to 12, to 24, to 48 h and beyond one week or as the postoperative day (POD) 0, 1, and 2 described in original studies.

### Trial sequential analysis

We performed Trial Sequential Analysis (TSA) using the TSA program (www.ctu.dk/tsa.) on the three critical outcomes (pain at rest, pain at ambulation, morphine consumption). TSA tests the credibility of the results by combining the estimation of information size (a cumulative sample size of included RCTs) with an adjusted threshold of statistical significance for the cumulative meta-analysis. The required information size (RIS) and meta-analysis monitoring boundaries (Trial Sequential Monitoring Boundaries) were quantified, alongside adjusted 95% confidence intervals. Diversity adjustment was performed according to an overall type I error of 5% and power of 80%.

### Meta-regression

High heterogeneity not fully explained by subgroup analysis was further investigated with a post hoc mixed-model meta-regression on the primary outcome (pain at ambulation). To avoid overfitting, meta-regression was performed only in the following clinically meaningful and explanatory variables: patient number, the multimodal analgesia protocol, types of other nerve blocks, anesthesia drug.

### Risk of bias assessment and publication bias

The methodology quality was independently evaluated by two reviewers using the Cochrane Collaboration’s Risk of Bias Tool [19]. The following domains were assessed and evaluated: randomization process, deviation from intended interventions, missing outcome data, measurement of outcomes, and selection of reported results. Each domain can be judged as low risk of bias, high risk of bias, or unclear, and overall risk of bias is expressed on a three-grade scale (low risk of bias, high risk of bias or unclear).

The funnel plots were used to assess publication bias when the included studies were more than 10 in the outcome, and the Egger test was further performed (when visual asymmetry was observed).

### Quality of evidence

We used the Grading of Recommendations Assessment, Development and Evaluation (GRADE) system to assess the certainty of the evidence in key outcomes. Study design, risk of bias, imprecision, inconsistency, indirectness, and magnitude of effect were considered. The level of evidence could be divided into four degrees: high, moderate, low, and very low. The rules for downgrade evidence were referenced in Guyatt’s studies [20–25]. We defined the following as critical outcomes: pain at ambulation, pain at rest, morphine consumption amount, the rate of rescue morphine use.

### Statistical analysis

Weight mean difference (WMD) for continuous variables (Mantel–Haenszel method) and risk ratios (RR) for dichotomous variables (inverse variance method) with 95% confidence intervals (95% CIs) were used. P values of < 0.05 were considered statistically significant. A random-effect model was used in the study. The heterogeneity was reported by I^2^ statistics. (I^2^ > 70% was considered as high heterogeneity.) Sensitivity analysis will be applied to examine the effect of deleting one single study on the overall estimate when observed high heterogeneity, and Publication bias was evaluated both by a visual inspection of funnel plots and by Egger test (*p* < 0.05 indicating a possible publication bias) using Egger’s regression intercept to quantify publication bias. The Review Manager 5.3 was used for drafting figures of risk of bias, and STATA 13.0 was used for data analysis.

## Results

### Study selection, data retrieval, and characteristics

**Fig. 1:**
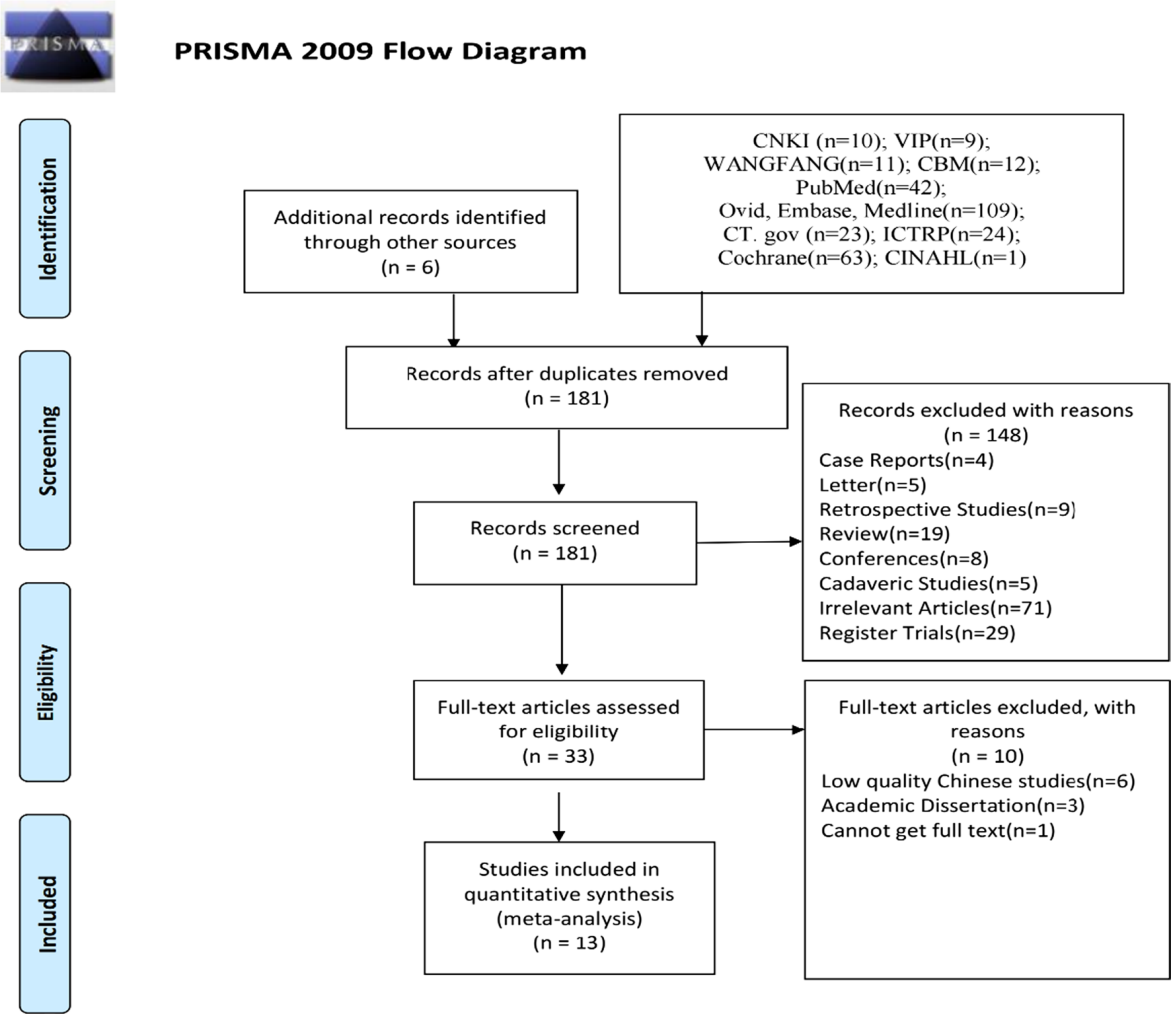
PRISMA flow diagram describing the selection process of studies

Our search initially yielded 310 potentially relevant papers and 181 articles remaining after duplicates. After title and abstract screening, 33 relevant papers were identified and remained full-text selection (Fig. 1). After reading the full text, we included 13 RCTs with 1347 patients (675 with IPACK; 672 without IPACK) [3, 7–16, 26, 27]. The overall analysis is summarized in Table 1. The sample size ranged from 56 to 120 patients. All studies were published between 2018 and 2020, and the mean follow-up period ranged from 2 days to 3 months. A detailed description of all included studies can be found in Tables 2 and 3. More confounding information can be found in Table 3.

**Table 1:** The results of meta-analysis

Variables	N (comparisons)	N (IPACK)	N (non-IPACK)	Pooled data		Heterogeneity	
				WMD/RR (95% CI)	P	I^2^ (%)	Ph
*Pain scores*							
Pain at rest, overall	47	2121	2131	− 0.489 (− 0.736, − 0.242)	< 0.0001*	94.2%	< 0.0001
By subgroup (Follow-up time)							
Pain at rest (2–4 h)	6	241	238	− 0.792 (− 1.786, 0.202)	0.119	95.8%	< 0.0001
Pain at rest (6–12 h)	13	656	653	− 0.960 (− 1.467, − 0.454)	< 0.0001*	95.7%	< 0.0001
Pain at rest (16–24 h)	10	475	477	− 0.224 (− 0.787, 0.339)	0.436	96.0%	< 0.0001
Pain at rest (32–48 h)	13	626	638	− 0.115 (− 0.389, 0.159)	0.410	92.6%	< 0.0001
Pain at rest (> 1w)	4	123	125	− 0.319 (− 0.621, − 0.016)	0.039*	0%	0.912
Pain at ambulation, overall	52	2047	2062	− 0.487 (− 0.719, − 0.255)	< 0.0001*	92.4%	< 0.0001
By subgroup (Follow-up time)							
Pain at ambulation (2–4 h)	9	349	348	− 0.483 (− 0.958, − 0.008)	0.046*	90.3%	< 0.0001
Pain at ambulation (6–12 h)	13	612	611	− 0.691 (− 1.064, − 0.318)	< 0.0001*	91.1%	< 0.0001
Pain at ambulation (24 h)	8	345	344	− 0.508 (− 1.273, 0.258)	0.194	94.6%	< 0.0001
Pain at ambulation (36–48 h)	10	410	422	− 0.203 (− 0.811, 0.404)	0.512	95.1%	< 0.0001
Pain at ambulation (> 1w)	12	371	377	− 0.586 (− 0.951, − 0.220)	0.002*	65.2%	0.001
*Morphine consumption*							
Oral morphine consumption (overall)	34	1296	1292	− 2.559 (− 4.625, − 0.494)	0.015*	62.0%	< 0.0001
By subgroup (Follow-up time)							
Morphine consumption (0–12 h)	7	273	269	− 2.019 (− 9.989, 5.950)	0.619	63.8%	0.040
Morphine consumption (12–24 h)	10	406	401	− 4.936 (− 11.517, 1.646)	0.142	75.7%	< 0.0001
Morphine consumption (24–48 h)	10	405	407	− 2.979 (− 5.714, − 0.244)	0.033*	0%	0.441
Morphine consumption (48− 72 h)	4	212	215	− 0.579 (− 2.892, 1.734)	0.624	61.4%	0.051
Morphine requirement (overall)	13	465	458	0.918 (0.635, 1.328)	0.649	45.2%	0.039
By subgroup (Follow-up time)							
Morphine requirement (0–12 h)	2	66	64	0.813 (0.377, 1.755)	0.599	0.0%	0.608
Morphine requirement (12–24 h)	4	142	139	0.506 (0.309, 0.829)	0.007*	3.8%	0.374
Morphine requirement (24–48 h)	5	157	155	0.841 (0.626, 1.131)	0.252	0.0%	0.825
Morphine requirement (48–72 h)	2	32	14	2.336 (0.953, 5.730)	0.064	54.3%	0.139
*Functional outcomes*							
ROM (Overall)	11	400	397	1.090 (− 3.740, 5.921)	0.658	90.2%	< 0.0001
By subgroup (Follow-up time)							
ROM (POD0)	1	34	35	− 2.700 (− 7.959, 2.559)	0.314	N/A	N/A
ROM (POD1)	4	160	159	1.002 (− 6.683, 8.687)	0.798	87.1%	< 0.0001
ROM (POD2)	4	140	139	4.221 (− 4.816, 13.258)	0.360	92.2%	< 0.0001
ROM (POD3)	2	66	64	− 3.200 (− 7.180, 0.780)	0.115	0%	1.000
TUG (Overall)	18	830	821	− 0.735 (− 3.352, 1.881)	0.582	74.6%	< 0.0001
By subgroup (Follow-up time)							
TUG (POD0)	1	34	35	− 18.60 (− 45.428, 8.228)	0.174	N/A	N/A
TUG (POD1)	3	127	126	− 4.901 (− 15.554, 5.753)	0.367	19.2%	0.290
TUG (POD2)	5	238	236	− 1.701 (− 9.572, 6.170)	0.672	91.8%	< 0.0001
TUG (POD3)	4	166	164	− 0.585 (− 5.641, 4.471)	0.821	42.8%	0.154
TUG (> 1w)	6	232	228	− 0.260 (− 1.812, 1.293)	0.743	0.0%	0.523
Ambulation distance (Overall)	15	662	660	1.122 (0.365, 1.878)	0.004*	0%	0.869
By subgroup (Follow-up time)							
Ambulation distance (POD0)	2	75	77	3.503 (− 6.804, 13.810)	0.505	0%	0.722
Ambulation distance (POD1)	6	266	265	0.798 (− 0.122, 1.718)	0.089	0%	0.633
Ambulation distance (POD2)	5	221	218	1.743 (0.339, 3.147)	0.015*	0%	0.563
Ambulation distance (POD3)	2	100	100	2.013 (− 2.476, 6.503)	0.379	0%	0.610
*QMS*							
By subgroup (Flexion Degrees)							
QMS, 0 degree (Overall)	21	769	761	0.405 (0.042, 0.767)	0.029*	94.4%	0.029
QMS, 45 degree (Overall)	11	342	342	0.146 (− 0.200, 0.492)	0.408	0.0%	0.796
QMS, 90 degree (Overall)	11	340	339	0.130 (− 0.268, 0.529)	0.521	0.0%	0.994
*Complications*							
PONV	4	173	172	0.920 (0.676, 1.252)	0.596	46.6%	0.132
*Perioperative outcomes*							
LOS	7	262	261	− 3.182 (− 6.568, 0.204)	0.066	64.2%	0.010
Operation time	12	574	568	− 0.241 (− 1.514, 1.032)	0.711	0%	0.940
Patients satisfaction	5	199	196	0.471 (− 0.015, 0.956)	0.058	88.4%	< 0.0001
Sleep disturbance (Overall)	12	399	393	0.499 (0.311, 0.799)	0.004*	10.6%	0.341
By subgroup (Follow-up time)							
Sleep disturbance (POD0)	4	133	131	0.505 (0.182, 1.405)	0.191	53.9%	0.089
Sleep disturbance (POD1)	4	133	131	0.388 (0.185, 0.812)	0.012*	0.0%	0.464
Sleep disturbance (POD2)	4	133	131	0.527 (0.190, 1.467)	0.220	0.0%	0.513

*ROM* range of motion, *TUG* time up and go, *QMS* quadriceps muscle strength, *PONV* postoperative nausea and vomiting, *LOS* length of operation, *POD* postoperative day

**Table 2:** The baseline characteristics

Study	Country	Period	Comparison		No. of Patients		Age^†^ (years)		Women^‡^ (no. [%])	
			IPACK	Non-IPACK	IPACK	Non-IPACK	IPACK	Non-IPACK	IPACK	Non-IPACK
El-Emam2020	Egypt	N/A	IPACK + SACB	SACB	28	28	52 (15)	54 (13)	8 (28.57%)	9 (32.14%)
Hu2020	China	N/A	IPACK + SACB	SACB	40	40	74.7 (6.3)	73.9 (4.9)	N/A	N/A
Kim2019	America	2017.03–2017.10	IPACK + SACB + mPAI	PAI	43	43	68.3 (7)	67.1 (8.1)	23 (53.48%)	30 (69.77%)
Kertkiatkachorn2020	Thailand	2019.05–2019.11	IPACK + SACB + CACB	CACB + PAI	38	38	70.6 (6.9)	68.7 (8.5)	29 (85.29%)	29 (82.85%)
Kampitak2020(Comparison A)	Thailand	2018.02–2019.01	Proximal IPACK + CACB	TNB + CACB	33	32	68.6 (6.1)	68.8 (6.5)	28 (84.84%)	28 (87.5%)
Kampitak2020(Comparison B)	Thailand	2018.02–2019.01	Distal IPACK + CACB	TNB + CACB	33	32	69.9 (6.6)	68.8 (6.5)	27 (81.8%)	28 (87.5%)
Li2019	China	2017.11–2018.04	IPACK + SACB	SACB	30	30	66 (6)	69 (6)	21 (70%)	16 (53.33%)
Li2020(Comparison A)	China	2018.05–2019.04	IPACK + SACB + LFCNB	SACB + LFCNB	50	50	66.26 (4.69)	66.40 (6.42)	33 (66%)	32 (64%)
Li2020(Comparison B)	China	2018.05–2019.04	IPACK + SACB	SACB	50	50	66.82 (6.17)	65.56 (6.34)	40 (80%)	31 (62%)
Ochroch2020	America	2018.11–2019.07	IPACK + CACB	CACB	60	59	67.7 (7.8)	65.6 (8.2)	34 (57%)	35 (60%)
Patterson2020	America	2016.11–2018.01	IPACK + CACB	CACB	35	34	67 (3.511)	68 (3.476)	21 (60%)	21 (62%)
Sankineani2018	India	2016.09–2017.03	IPACK + SACB	SACB	60	60	60	61	38 (63.33%)	42 (70%)
Tak2020(Comparison A)	India	2019.03–2019.06	IPACK + SACB	CACB	56	57	65.5	63.3	29 (51.8%)	38 (66.7%)
Tak2020(Comparison B)	India	2019.03–2019.06	IPACK + SACB	SACB	56	57	65.5	64.1	29 (51.8%)	37 (63.8%)
Vichainarong2020	Thailand	2018.07–2019.05	IPACK + CACB + LIA	CACB + LIA	33	32	70.7 (8.2)	68.7 (7.9)	29 (87.87%)	27 (84.37%)
Zheng2020	China	N/A	IPACK + SACB	FNB + SNB	30	30	62 (6)	61 (7)	21 (63.64%)	20 (66.66%)

Study	Country	Period	Women^‡^ (no. [%])		BMI^†^ (kg/m2)		Inclusion	Exclusion	Conclusion
			IPACK	Non-IPACK	IPACK	Non-IPACK			
El-Emam2020	Egypt	N/A	8 (28.57%)	9 (32.14%)	29.1 (2.7)	28.5 (3)	Age > 45 years; ASA I–III; Be competent to understand the study protocol; Radiographic evidence of OA (> Grade II); Chronic pain for at least 6 months; Conservative therapies were useless during the last 6 months;	Patient refusal; Bleeding or coagulation disorders; Local skin infection or any other medical problem in the affected limb; Psychiatric problems lead to difficult communication with the patients; Previous chronic opioid use; Contraindications to steroid injection as diabetes or hypertension	Combined SACB and IPACK block provide more effective analgesia and better functional outcomes compared to the SACB alone
Hu2020	China	N/A	N/A	N/A	21.2 (1.9)	20 (4.2)	Age between 65 to 89; ASA I-III; BMI18.5–23.7 kg/m^2^; Selective unilateral primary TKA;	Severe cardiovascular disease; Severe pulmonary dysfunction; Diseases of the central nervous system; Fail to communicate and cooperate; Coagulation disorders; Puncture site infection; Allergic to local anesthetic drugs;	IPACK group decreased postoperative remedial analgesia and the use of vasoactive drugs, but the postoperative VAS scores are similar after 24 h and 48 h; Ultra-guided IPACK and ACB are safe and effective in old patients with primary TKA;
Kim2019	America	2017.03–2017.10	23 (53.48%)	30 (69.77%)	28.3 (4.1)	29.9 (4.8)	Patients with OA scheduled for primary unilateral TKA with a participating surgeon; Age 18–80 years old, planned use of regional anesthesia, able to follow study protocol, and English speaking;	Hepatic or renal insufficiency, age < 18 or > 80 years old, patients undergoing general anesthesia, allergy or intolerance to one of the study medications, BMI > 40, diabetes mellitus, ASA IV, chronic gabapentin or pregabalin use (regular use for > 3 months), chronic opioid use (taking opioids for > 3 months, or daily oral morphine equivalent of > 5 mg/d for 1 month), and patients with severe valgus deformity and flexion contracture	We conclude that the addition of IPACK and ACB to PAI for pain management in TKA patients improves postoperative pain, opioid consumption, and measures of pain-related patient satisfaction
Kertkiatkachorn2020	Thailand	2019.05–2019.11	29 (85.29%)	29 (82.85%)	27.2 (3.8)	28 (4.2)	Ages between 18 and 80 years; ASA I to III; Scheduled to undergo the first two elective TKAs of the day were screened for eligibility	Exclusion criteria were a varus-valgus deformity of > 20°, knee flexion deformity > 30°, known allergy to the drugs used in this trial, body mass index < 18 or > 40 kg/m2, contraindication for neuraxial or regional anesthesia, contraindication for NSAIDs, chronic opioid use (defined as a history of regular opioid use for more than 3 months or a history of oral morphine use equivalent of > 60 mg/month), failure to perform the Timed Up and Go test and, inability to communicate or unwilling to give informed consent	The combination of ACB and IPACK block provides noninferior analgesia compared with PAI when combined with CACB during part of postoperative multimodal analgesia regimens for patients undergoing TKA. However, the ACB + IPACK block may be associated with a higher level of opioid consumption and lower ambulatory ability on the day of surgery
Kampitak2020(Comparison A)	Thailand	2018.02–2019.01	28 (84.84%)	28 (87.5%)	27.6 (4.2)	28.6 (3.9)	Inclusion criteria were age > 18 years; ASA I–III; BMI:18–40 kg/m2	Exclusion criteria were inability to cooperate, allergy to any drug administered in this study, contraindications to neuraxial and/or regional anesthesia, lower limb neuropathy involving the operative site, intolerance to non-steroidal anti-inflammatory drugs, chronic opioid drug use (daily or almost daily use of opioid drugs for at least 3 months, or morphine use greater than or equal to 60 mg/day for at least 1 month, or diagnosis of neuropathic pain), and inability to perform the timed up- and- go (TUG) test	Distal IPACK block were better able to preserve the normal motor function of the common peroneal nerve and tibial nerve compared with those who received the proximal IPACK block or TNB;
Kampitak2020(Comparison B)	Thailand	2018.02–2019.01	27 (81.8%)	28 (87.5%)	26.3 (3.8)	28.6 (3.9)	See in Kampitak2020 (Comparison A)	See in Kampitak2020 (Comparison A)	See in Kampitak2020 (Comparison A)
Li2019	China	2017.11–2018.04	21 (70%)	16 (53.33%)	21.9 (2.2)	21.7 (2)	Primary unilateral TKA; Age between 55 to 78 years; ASA I-III;	Severe cardiovascular disease; Severe pulmonary dysfunction; Diseases of the central nervous system; Fail to communicate and cooperate; Coagulation disorders; Puncture site infection; Allergic to local anesthetic drugs;	IPACK plus SACB added to multimodal analgesic methods could provide satisfied effect
Li2020(Comparison A)	China	2018.05–2019.04	33 (66%)	32 (64%)	24.82 (2.58)	24.81 (3.15)	Aged between 50 and 80 years; BMI 19–30 kg/m2; ASA I-III; Scheduled to have primary unilateral TKA for osteoarthritis;	Exclusion criteria included the following: (1) knee flexion deformity ≥ 30°, varus-valgus deformity ≥ 30°, and inability to walk; (2) allergy to morphine or had a past history of opioid consumption; (3) had any contraindications to regional anesthesia, local infiltration, general anesthesia, and the drugs used in this study; (4) diagnosis of septic arthritis, rheumatic arthritis, traumatic arthritis, and other non-OA; and (5) patients with a medical history of psychiatric illness, cognitive impairment, recognized neuromuscular disorder, narcotic dependency, knee infection, knee surgery, or thromboembolic event including myocardial infarction, cerebrovascular accident, deep vein thrombosis, and pulmonary embolus. Additionally, patients with a language barrier, or those who refused to sign informed consent, were also excluded	ACB with IPACK block and LFCNB may decrease the early postoperative pain scores and prolong analgesic duration following TKA. Compared to ACB with IPACK, ACB with LFCNB, or ACB alone, this method produced optimal outcomes without increased complications
Li2020(Comparison B)	China	2018.05–2019.04	40 (80%)	31 (62%)	24.68 (2.60)	24.97 (3.18)	see in Li (Comparison A)	see in Li (Comparison A)	see in Li (Comparison A)
Ochroch2020	America	2018.11–2019.07	34 (57%)	35 (60%)	31.9 (6.4)	31.3 (7.0)	Patients with ASA I-III undergoing primary TKA; Age 18–80 years;	Patients were excluded from the study if they had an allergy to any of the study medications, BMI > 45, coagulopathy, chronic kidney disease or recent chronic opioid therapy, defined as the use of regular daily doses of systemic opioids for the past 3 months prior to the surgery. Revision knee replacement procedures were also excluded	IPACK block reduced the incidence of posterior knee pain 6 h postoperatively. Given the relative ease and safety profile, it may have a potential role as part of the multimodal analgesia after knee arthroplasty, particularly as a distinct alternative to sciatic nerve blockade that does not affect motor function. The IPACK block can also be considered as a more consistent and reproducible alternative to surgical PAI of the posterior capsule of the knee, but more studies are needed
Patterson2020	America	2016.11–2018.01	21 (60%)	21 (62%)	31 (1.732)	30 (1.450)	Eligible patients with elective unilateral, primary TKA; Age > 18 years old; English speaking; ASAI-III	Exclusion criteria were contraindication to regional anesthesia or peripheral nerve blocks, allergy to local anesthetics, nonsteroidal anti-inflammatory drugs (NSAIDs), chronic renal insufficiency (Cr > 1.4 mg/dL or glomerular filtration rate < 60 mL/min), chronic pain not related to the knee joint, chronic opiate consumption (daily or almost daily use for ≥ 3 months), pre-existing peripheral neuropathy involving the operative site, and body mass index > 40 kg/m2	IPACK and CACB improved pain scores in the immediate postoperative period but otherwise provided no additional benefit in pain scores, opioid consumption, physical therapy performance, the frequency of opioid-related side effects, and hospital length of stay were not affected by the addition of the IPACK. Therefore, IPACK and CACB may not provide a significant clinical benefit in TKA patients
Sankineani2018	India	2016.09–2017.03	38 (63.33%)	42 (70%)	29.36	28.88	N/A	Patients undergoing bilateral or revision total knee replacement, with history of bleeding diathesis or prior vascular surgery on femoral vessels on operated site, severe renal insufficiency, history of arrhythmia or seizures, sepsis, preexisting lower extremity neurological abnormality and difficulties in comprehending visual analog scale (VAS) pain scores, were excluded from the study	ACB + IPACK is a promising technique that offers improved pain management in the immediate postoperative period without affecting the motor function around the knee joint resulting in better ROM and ambulation compared to ACB alone
Tak2020(Comparison A)	India	2019.03–2019.06	29 (51.8%)	38 (66.7%)	26	26	Unilateral tricompartmental TKA for primary OA; Age 45–80 years; ASA I–III	Exclusion criteria included patients who underwent bilateral or revision TKA, knee flexion deformity of ≥ 30°, varus–valgus deformity of ≥ 30°, arthritis due to rheumatoid disease or trauma or septic arthritis, creatinine > 1.2, renal or hepatic dysfunction, known allergy to any study medication, chronic opioid use, BMI > 40, chronic pain unrelated to knee joint, pre-existing neuropathy, arrhythmia, epilepsy, had a history of bleeding diathesis or prior vascular surgery on femoral vessels on operated site and difficulty in comprehending VAS pain scores	CACB provides better pain control, decreased opioid consumption and superior ambulation capacity in the immediate postoperative period compared to SACB + IPACK without any significant adverse side effects
Tak2020(Comparison B)	India	2019.03–2019.06	29 (51.8%)	37 (63.8%)	26	26.6	See in TAK (comparison A)	See in TAK (comparison A)	This study also concludes that the addition technique of IPACK to SACB may not add any additional benefit in postoperative pain control, ambulation, opioid consumption or rehabilitation compared to SACB alone
Vichainarong2020	Thailand	2018.07–2019.05	29 (87.87%)	27 (84.37%)	27 (4.4)	28.2 (4.2)	Adult patients with ASA I–III scheduled for elective primary TKA using standard spinal anesthesia	Age < 18 or > 80 years; BMI > 40 kg/m^2^; inability to provide informed consent; cognitive or psychiatric history that may interfere with assessment; a varus-valgus knee deformity > 20°; knee flexion deformity > 30°; contraindication for spinal anesthesia or peripheral nerve block; allergy or intolerance to local anesthetic drugs or any component of the multimodal analgesic regimen; pre-existing chronic pain or opioid drug use (daily or almost daily use of opioid drugs for ≥ 3 months or morphine use ≥ 60 mg/day for ≥ 1 month); Pre-existing neuropathy or neurological deficit in the lower extremities	The addition of an IPACK block to the LIA and CACB does not reduce the postoperative opioid consumption nor improve analgesia. However, it may improve immediate functional performance and reduce the length of hospitalization after TKA
Zheng2020	China	N/A	21 (63.64%)	20 (66.66%)	27.1 (3.4)	26.7 (2.7)	Age between 18 to 65 years; BMI between 18–24 kg/m^2^; ASA I or II;	Infection diseases; Nerve damage on operation side; Coagulation dysfunction; Liver or kidney diseases; Analgesic allergy; mental disfunction	IPACK and SACB could help improve the postoperative function recovery

*IPACK* interspace between the popliteal artery and capsule of the knee, *SACB* single abductor canal block, *CACB* continues abductor canal block, *ASA* American Society of Anesthesiologists, *OA* osteoarthritis, *BMI* body mass index, *TKA* total knee arthroplasty, *VAS* visual analogue scale, *mPAI* modified periarticular injection, *TNB* tibial nerve block, *LFCNB *lateral femoral cutaneous nerve block, *LIA *local infiltration anesthesia, *SNB* sciatic nerve block

^†^The values are presented as the mean and the standard deviation

^‡^The values are given as the number of patient and the percentage of the group

**Table 3:** The confounding factors of included studies

Study	Country	ASA	Medications		Multi-modal Pain Management Methods				
			IPACK	Non-IPACK	Rescue Methods	Anesthesia	Pre-operative	Intra-operative	Post-operative
El-Emam2020	Egypt	I/II:50/6	(IPACK + SACB) SACB: 10 mL of 0.125 bupivacaine plus 40 mg methylprednisolone IPACK: 10 mL of 0.125 bupivacaine plus 40 mg methylprednisolone;	(SACB) SACB: 10 mL of 0.125 bupivacaine plus 40 mg methylprednisolone	N/A	N/A	N/A	N/A	N/A
Hu2020	China	I/II/III: 25/39/16	(IPACK + SACB) IPACK: 0.2% ropivacaine 15 ml SACB:0.2% ropivacaine 20 ml;	(SACB) SACB:0.2% ropivacaine 20 ml;	VAS > 5, 20–40 mg Parecoxib sodium was given via Intravenous injection	General anesthesia	N/A	Propofol 3–5 mg/(kg h), Remifentanil 10–15 g/ (kg h) and other medications were adjusited by patients' situation	PCA: the analgesic formula was sufentanil 2 μg/kg, dezocine 10 mg, and Ondansetrone 16 mg + 0.9% sodium chloride injection diluted to 100 ml, the basic dose was 2 ml/h, the additional dose was 2 ml/time, and the locking time was 15 min
Kim2019	America	I/II/III: 1/81/4	IPACK + SACB + mPAI IPACK: 25 mL of 0.25% bupivacaine; SACB: 15 mL of bupivacaine 0.25% with 2 mg of preservative-free dexamethasone; mPAI: bupivacaine 0.25% with 1:300,000 epinephrine at a volume of 30 mL; methylprednisolone, 40 mg/mL in 1 mL; cefazolin, 500 mg in 10 mL; and normal saline, 22 ml; note:mPAI: modified PAI	PAI PAI: bupivacaine 0.5% with 1:300,000 epinephrine at a volume of 30 Ml, methylprednisolone, 40 mg/mL in 1 mL; cefazolin, 500 mg in 10 mL; and normal saline, 22 mL; 20 mL of 0.25% bupivacaine; 2 mg IV dexamethasone and ensure 10 mg dexamethasone via all route	NRS > 6 for 2 h, an IV hydromorphone PCA was ordered	spinal epidural anesthetic	Meloxicam: 7. 5 mg per os if age ≥ 75 or older 15 mg otherwise; Extended-release oxycodone (10 mg per os) in the holding area	Combined spinal epidural anesthetic with 60 mg mepivcaine spinal IV sedation: 2–5 mg with midazolam and propofol infusion; Ondansetron: 4 mg IV Famotidine: 20 mg IV Fentanyl: up to 100 mcg;	1. Acetaminophen: 1000 mg IV every 6 h for 4 doses. Then, 1 g PO every 8 h 2. Ketorolac: 30 mg IV every 6 h for 4 doses. If patient is 75 or older, 15 mg IV every 6 h for 4 doses 3. Oxycodone (IR): 5 mg (for NRS pain 0–4) or 10 mg (for NRS 5–10) every 3 h PRN; 4. Meloxicam: 15 PO to start after ketorolac is finished (7.5 mg PO if age > 75 years old); 5. Hydromorphone: 0. 5 mg IV every 10 min × 4 doses for breakthrough pain (NRS > 6,rescue analgesia);
Kertkiatkachorn2020	Thailand	I/II/III: 3/58/6	(IPACK + SACB + CACB) IPACK: 20 mL of 0.25% levobupivacaine with ketorolac (15 mg) and epinephrine (0.1 mg) SACB: 20 mL of 0.25% levobupivacaine with ketorolac (15 mg) and epinephrine (0.1 mg) with intermittent negative aspirations CACB: 0.15% levobupivacaine (5 mL/h for 60 h)	CACB + PAI CACB: 0.15% levobupivacaine (5 mL/h for 60 h) PAI: 20 mL of 0.5% levobupivacaine, 30 mg of ketorolac, 0.3 mg of epinephrine combined with isotonic saline for a total volume of up to 80 mL into the posterior capsule, medial and lateral collateral ligament insertions, medial and lateral meniscus remnant, anterior capsule, suprapatellar pouch, fat pad, and soft tissue;	VAS score ≥ 4 during their stay in PACU, 2 mg of IV morphine was administered every 30 min	spinal anesthesia(3 mL of 0.5% hyperbaric bupivacaine without intrathecal morphine)	All patients received oral acetaminophen (2 × 375-g tablets) and oral celecoxib (400-g tablet) 30 min before surgery	Dexamethasone (10 mg) and ondansetron (4 mg) were administered for postoperative nausea and vomiting prophylaxis	Parecoxib (40 mg IV every 12 h; 2 doses) Acetaminophen (orally, 650 mg per dose every 6 h) Pregabalin (orally, 75 mg per dose once a day), and Celecoxib (orally, 400 mg per dose once a day; started after the last dose of parecoxib)
Kampitak2020(Comparison A)	Thailand	I/II/III: 1/62/2 1	(Proximal IPACK + CACB) Proximal IPACK: 5 mL 0.25% levobupivacaine with 1:200,000 epinephrine; simultaneously, the needle was slowly withdrawn, and 15 mL of local anesthetic was injected until the tip of the needle reached the end of the medial aspect of the femur CACB: 15 mL of 0.25% levobupivacaine was injected with intermittent negative aspirations, 0.15% levobupivacaine was continuously dripped at 5 mL/hour via a disposable infusion pump LIA: 20 mL of 0.5% levobupivacaine, 0.3 mL of 1:1000 epinephrine, 30 mg of ketorolac, and 40 mL of isotonic sodium chloride solution;	TNB + CACB TNB:15 ml 0.25% levobupivacaine were injected in divided doses of 5 mL, aspirating frequently to avoid intravascular injection CACB: same with intervention group LIA: same with intervention group	NRS > 4, 2 mg of intravenous morphine was administered every 30 min; Continued NRS > 4 for up to 1 h, PCA was administered using Morphine (no basal rate, PCA dose 2 mg, lockout 10 min);	spinal anesthesia (3 mL of 0.5% hyperbaric bupivacaine)	Lorazepam (0.5 mg) was administered orally on the night before surgery(mild or worse anxiety); Paracetamol (650 mg orally) 30 min prior to surgery as premedication;	Intravenous dexamethasone (10 mg) and ondansetron (4 mg) for postoperative nausea and vomiting prophylaxis	20 mg of intravenous parecoxib every 12 h on postoperative day (POD) 0–1; 650 mg of acetaminophen orally every 6 h; 75 mg of pregabalin orally once daily; After the last dose of parecoxib, 400 mg of celecoxib and half a tablet of tramadol hydrochloride/acetaminophen were administered, followed by 650 mg of acetaminophen orally every 6 h as needed
Kampitak2020(Comparison B)	Thailand	I/II/III: 1/62/2	(Distal IPACK + CACB) Distal IPACK: 20 mL of 0.25% levobupivacaine with 1:200 000 epinephrine was injected while slowly withdrawing the needle until the tip of the needle reached the medial femoral condyle; CACB: same with intervention group;	(TNB + CACB) TNB: same with intervention group CACB: same with intervention group	same as Comparison A	same as Comparison A	same as Comparison A	same as Comparison A	same as Comparison A
Li2019	China	I/II/III: 6/38/16	IPACK + SACB IPACK: 0.33% ropivacaine 15 ml SACB: 0.33% ropivacaine 20 ml;	SACB SACB: 0.33% ropivacaine 20 ml	NRS > 5, Nalbuphine was injected at 0.08 mg/kg(intravenously)	Combined spinal and epidural anesthesia(0.5% bupivacaine 1.6–2 ml, lidocaine was added as needed);	Flurbiprofen 50 mg(Intravenous injection)	N/A	Celecoxib 200 mg, bid, po
Li2020(Comparison A)	China	I/II/III: 17/52/31	IPACK + SACB + LFCNB SACB: 20 ml AV IPACK: 20 ml AV LFCNB: 10 ml AV LIA: 60 ml AV note: AV, 0.2% ropivacaine and 2.0 ug/ mL of epinephrine	SACB + LFCNB SACB: 20 ml AV IPACK: 20 ml placebo LFCNB: 10 ml AV LIA: 60 ml AV	Morphine hydrochloride (10 mg) was intramuscularly administered with untolerate pain reported by patients	N/A	N/A	Tranexamic acid (first dose of 20 mg/kg IV used during surgery; another dose used 8 h later); Elastic bandage to reduce the blood loss;	Postoperatively, ice compression devices were applied. Loxoprofen (60 mg, 1 tablet, b.i.d) was prescribed to control postoperative pain and alprazolam (0.4 mg, 1 tablet, qn) was given as a sleep aid; Tourniquet was used; After hospital discharge, patients were given rivaroxaban orally (10 mg, qd) to prevent venous thromboembolism for 2 weeks, loxoprofen orally for pain control (60 mg twice a day) until patients felt no pain, and were introduced to functional recovery methods
Li2020(Comparison B)	China	I/II/III: 22/43/35	IPACK + SACB SACB: 20 ml AV IPACK: 20 ml AV LFCNB: 10 ml placebo LIA: 60 ml AV	SACB SACB: 20 ml AV IPACK: 20 ml placebo LFCNB: 10 ml placebo LIA: 60 ml AV;	See in Li (comparison A)	See in Li (comparison A)	See in Li (comparison A)	See in Li (comparison A)	See in Li (comparison A)
Ochroch2020	America	I/II/III: 1/65/53	IPACK + CACB CACB: ropivacaine 0.2% at a basal rate of 8 mL/ hour with a PCA of 5 mL every 30 min; IPACK: 20 ml of ropivacaine 0.5%;	CACB CACB: ropivacaine 0.2% at a basal rate of 8 mL/ hour with a PCA of 5 mL every 30 min Sham IPACK: superficial injection of local anesthetic to create a skin weal of the medial side of the knee;	Spinal (99,75%)/General (30,25%); Spinal anesthesia: bupivacaine 10–15 mg; Ketamine 0. 3–0. 5 mg/kg intravenously;	Acetaminophen 1000 mg PO Gabapentin 300 mg PO Celecoxib 200 mg PO Adductor canal catheter, ropivacaine 0. 5%20 mL	All patients received prophylaxis for postoperative nausea and vomiting: including 4 mg of dexamethasone; 4 mg of ondansetron 20 min before recovery from anesthesia; (dexamethasone was withheld in patients with blood glucose above 250 mg/dL)	Adductor canal catheter, ropivacaine 0. 2%8 mL/hour with demand bolus of 5 mL, lockout interval 30 min in 2 days Acetaminophen 1000 mg PO every 8 h in 3 days Celecoxib 200 mg PO every 12 h in 3 days Gabapentin 300 mg PO every 12 h in 7 days Oxycodone 5–10 mg PO every 4 h per registered nurse;	N/A
Patterson2020	America	I/II/III: 3/44/22	IPACK + CACB CACB: 20 mL ropivacaine 0.25% with epinephrine 3 mcg/ml; 8 mL/h continuous infusion of ropivacaine 0.2% was initiated through the adductor canal catheter; IPACK: 15 ml ropivacaine 0.25% with epinephrine 3 mcg/mL with an additional 5 ml of local anesthesia, a total of 20 mL of local anesthetic	CACB CACB: 20 mL ropivacaine 0.25% with epinephrine 3 mcg/ml; 8 mL/h continuous infusion of ropivacaine 0.2% was initiated through the adductor canal catheter; sham IPACK: 2 ml 0.9% saline for sham IPACK;	Oxycodone immediate-release tablets, IV morphine, and/or IV hydromorphone were available for breakthrough pain not relieved by oral medications	Neuraxial block or general anesthesia	All patients received 150 mg pregabalin (75 mg for patients aged > 70 years)	Patients received intravenous (IV) ketamine 0.25 mg/kg (up to 50 mg) and dexamethasone 8 mg IV	Patients were prescribed 1 g IV acetaminophen followed by 1 g oral acetaminophen every 6 h while in the hospital, 400 mg oral celecoxib followed by 200 mg daily, and 75 mg or 150 mg oral pregabalin daily in the evening
Sankineani2018	India	N/A	IPACK + SACB IPACK: 15 ml of 0.2% ropivacaine SACB:20 ml of 0.2% ropivacaine;	SACB SACB:20 ml of 0.2% ropivacaine	If patients have breakthrough pain, Intravenous diclofenac 75 mg along with a transdermal buprenorphine patch (5 mcg/h)	Spinal anesthesia(2.5 ml 0.5% hyperbaric bupivacain)	N/A	N/A	Postoperative analgesic regimen: paracetamol 1 g intravenously every 8 h for 3 days followed by oral paracetamol 1 g every 8 h for 1 month, gabapentin 300 mg given orally once daily for a period of 4 weeks
Tak2020(Comparison A)	India	II/III: 106/7	IPACK + SACB SACB:0.2% ropivacaine 20 ml IPACK: 0.2% ropivacaine 20 ml	CACB CACB: 0.2% ropivacaine via catheter at 5 ml/h for 48 h	Oxycodone immediate release tablets or intravenous morphine was considered in the form of rescue analgesia	spinal anesthesia	oral celecoxib 200 mg and gabapentin 300 mg preoperatively 10 h before surgery	N/A	intravenous paracetamol 1 g was given every 8 h for 3 days followed by oral paracetamol 1 g every 8 h along with Gabapentin 300 mg given orally once daily for a period of 4 weeks
Tak2020(Comparison B)	India	II/III: 106/8	IPACK + SACB SACB:0.2% ropivacaine 20 ml IPACK: 0.2% ropivacaine 20 ml	SACB SACB:0.2% ropivacaine 20 ml	see in TAK(Comparison A)	see in TAK(Comparison A)	see in TAK(Comparison A)	see in TAK(Comparison A)	see in TAK(Comparison A)
Vichainarong2020	Thailand	I/II/III: 3/59/3	IPACK + CACB + LIA IPACK: 5 mL of 0.25% levobupivacaine with 1:200,000 epinephrine CACB: 20 mL 0.25% levobupivacaine, Levobupivacaine 0.15% was continuously dripped at 5 mL/hour via a disposable infusion pump for 60 h postoperatively LIA: levobupi vacaine 100 mg, ketorolac 30 mg, epinephrine 0.3 mg diluted with isotonic sodium chloride solution to a total volume of 80 mL;	CACB + LIA CACB: 20 mL 0.25% levobupivacaine, Levobupivacaine 0.15% was continuously dripped at 5 mL/hour via a disposable infusion pump for 60 h postoperatively LIA: levobupi vacaine 100 mg, ketorolac 30 mg, epinephrine 0.3 mg diluted with isotonic sodium chloride solution to a total volume of 80 mL;	If patients presented with persisting pain and NRS ≥ 4, the patient would receive 2 mg of intravenous morphine as rescue therapy	spinal anesthesia: 15 mg of 0.5% hyperbaric bupivacaine;	All patients received 650 mg of acetaminophen and 400 mg of celecoxib orally 30 min before surgery	All patients received 10 mg of dexamethasone and 4 mg of ondansetron intravenous for postoperative nausea and vomiting prophylaxis;	Two consecutive doses of 15 mg ketorolac intravenous, 650 mg oral acetaminophen every 6 h, and 75 mg oral pregabalin (Lyrica) daily; After the last dose of ketorolac intravenous, 400 mg oral celecoxib (Celebrex) daily and half a tablet of tramadol hydrochloride/acetaminophen (Ultracet) were administered every 8 h; 40 mg intravenous esomeprazole daily for preventing upper gastrointestinal bleeding and 4 mg intravenous ondansetron every 6 h to prevent nausea and vomiting
Zheng2020	China	I/II: 17/33	IPACK + SACB IPACK: 0.375% ropivacaine 15 ml SACB: 0.375% ropivacaine 25 ml;	FNB + SNB FNB: 0.375% ropivacaine 20 ml SNB: 0.375% ropivacaine 20 ml;	VAS > 3, Intravenous sufentanyl was used as 0.1 μg/kg	N/A	Intravenous Administration: Midazolam 0.02 mg/kg Sufentanil 0.2–0.3 g/kg Etomidate 0.2 mg/kg Aquarium sulfonate 0.6 mg/kg	Intravenous Administration: sufentanil 2 μg/kg; Ondansetron 8 mg and sterile saline all 100 ml; The background infusion rate is 2 ml/h and the lock time is 15 min	N/A

Study	Country	ASA	Surgical factors				ITT	Follow-up	Lost (n)
			Surgeons	Prothesis	Approach	Others			
El-Emam2020	Egypt	I/II:50/6	N/A	N/A	N/A	Only OA patients included	No	12w	0
Hu2020	China	I/II/III: 25/39/16	N/A	N/A	N/A	N/A	No	2d	0
Kim2019	America	I/II/III: 1/81/4	Coinvestigator surgeons	N/A	N/A	Tourniquet was used	Yes	2d	0
Kertkiatkachorn2020	Thailand	I/II/III: 3/58/6	performed by or under the supervision of two senior surgeons	tricompartmental prostheses; hand-mixed cementing techniques	minimally invasive minimidvastus approach	N/A	Yes	2 m	2
Kampitak2020(Comparison A)	Thailand	I/II/III: 1/62/2 1	Performed by three orthopedic surgeons	hand-mixed cementing techniques; tricompartmental prostheses	minimally invasive minimidvastus approach	Tourniquet was used	Yes	6w	5
Kampitak2020(Comparison B)	Thailand	I/II/III: 1/62/2	same as Comparison A	same as Comparison A	same as Comparison A	same as Comparison A	same as Comparison A	S same as Comparison A	same as Comparison A
Li2019	China	I/II/III: 6/38/16	senior surgeons	N/A	N/A	N/A	No	2d	0
Li2020(Comparison A)	China	I/II/III: 17/52/31	performed by 2 senior surgeons	Prostheses: DePuy P.F.C; Stryker Triathlon;	standard medial parapatellar approach	LIA was used in every group	No	3 m	0
Li2020(Comparison B)	China	I/II/III: 22/43/35	See in Li (comparison A)	See in Li (comparison A)	See in Li (comparison A)	See in Li (comparison A)	See in Li (comparison A)	see in Li (Comparison A)	see in Li (Comparison A)
Ochroch2020	America	I/II/III: 1/65/53	N/A	N/A	N/A	N/A	No	2w	1
Patterson2020	America	I/II/III: 3/44/22	Performed by one of three fellowship-trained total joint surgeons	posterior stabilized prosthesis	medial parapatellar approach	tourniquet was used	No	2d	2
Sankineani2018	India	N/A	performed by a single surgeon (AVGR)	Posterior stabilized knee prosthesis	medial parapatellar approach	All patients were discharged in POD 3	No	2d	0
Tak2020(Comparison A)	India	II/III: 106/7	Two fellowship trained joint replacement surgeons	Posterior stabilized knee prosthesis without patellar resurfacing	medial parapatellar approach	All patients had a standard supervised rehabilitation program and were discharged on POD 3; Adductor canal catheter was removed on POD2;	No	2d	0
Tak2020(Comparison B)	India	II/III: 106/8	see in TAK(Comparison A)	see in TAK(Comparison A)	see in TAK(Comparison A)	see in TAK(Comparison A)	No	2d	0
Vichainarong2020	Thailand	I/II/III: 3/59/3	performed by or under the supervision of two senior surgeons	minimally invasive minimidvastus approach	N/A	N/A	Yes	2 m	1
Zheng2020	China	I/II: 17/33	N/A	N/A	N/A	N/A	No	2d	0

* represented a significant difference, indicating* p* < 0.05

*IPACK* interspace between the popliteal artery and capsule of the knee, *SACB* single abductor canal block, *CACB* continues abductor canal block, *ASA* American Society of Anesthesiologists, *OA* osteoarthritis, *BMI* body mass index, *TKA* total knee arthroplasty, *VAS* visual analogue scale, *mPAI* modified periarticular injection, *TNB* tibial nerve block, *LFCNB* lateral femoral cutaneous nerve block, *LIA* local infiltration anesthesia, *SNB* sciatic nerve block

### Methodological quality

**Fig. 2:**
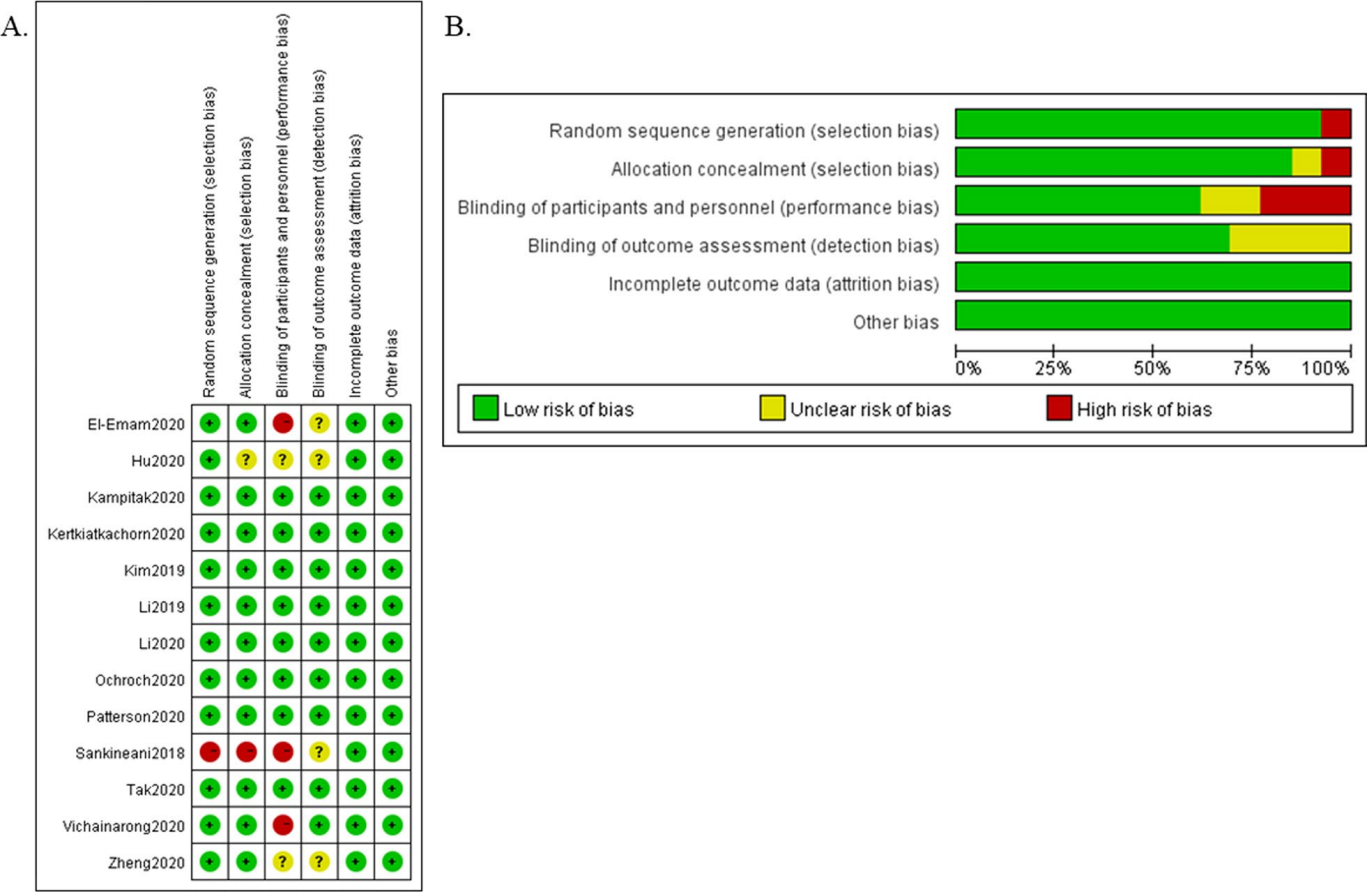
Risk of bias **a** risk of bias graph. **b** Risk of bias summary

According to the risk of bias evaluation, twelve studies clearly described randomization methods except one [27]. In eleven studies, appropriate methods were used to describe allocation concealment [3, 7–13, 15, 16, 26]. Blinding of the participants and personnel in eight studies was well described [3, 7, 10–13, 15, 16, 26]. The blinding of outcome assessors in nine studies was well performed [3, 7, 9–12, 15, 16, 26]. The proportion of patients lost to follow-up was less than 10% in all studies, indicating low attrition bias. All studies reported satisfactory outcomes, and the risk of reporting bias was low. No other bias was detected. The risk of bias overall and in each domain can be seen in Fig. 2.

### Pain scores at ambulation

**Fig. 3:**
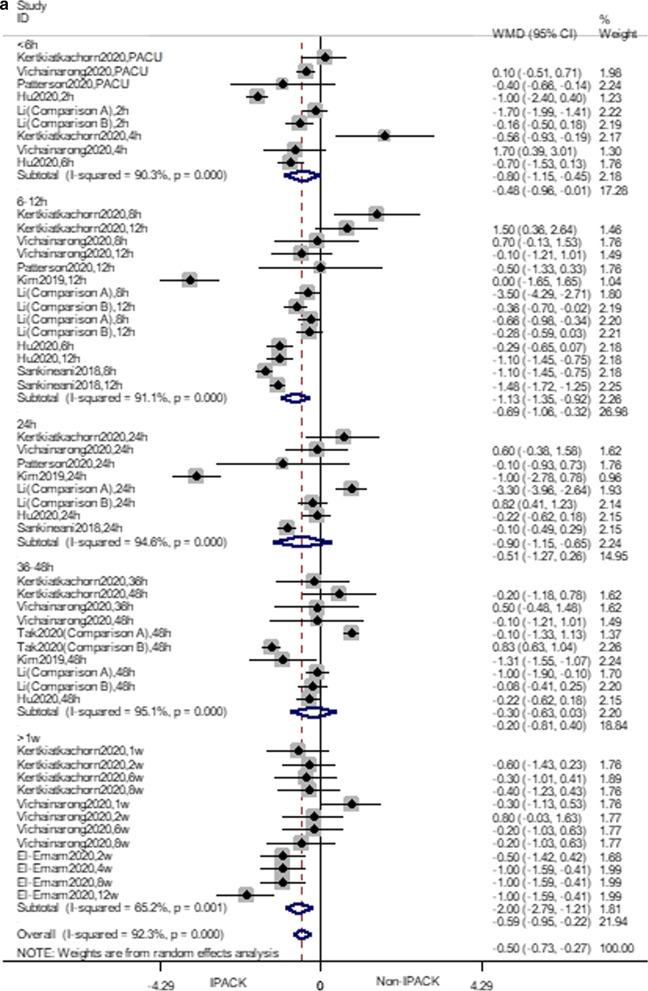

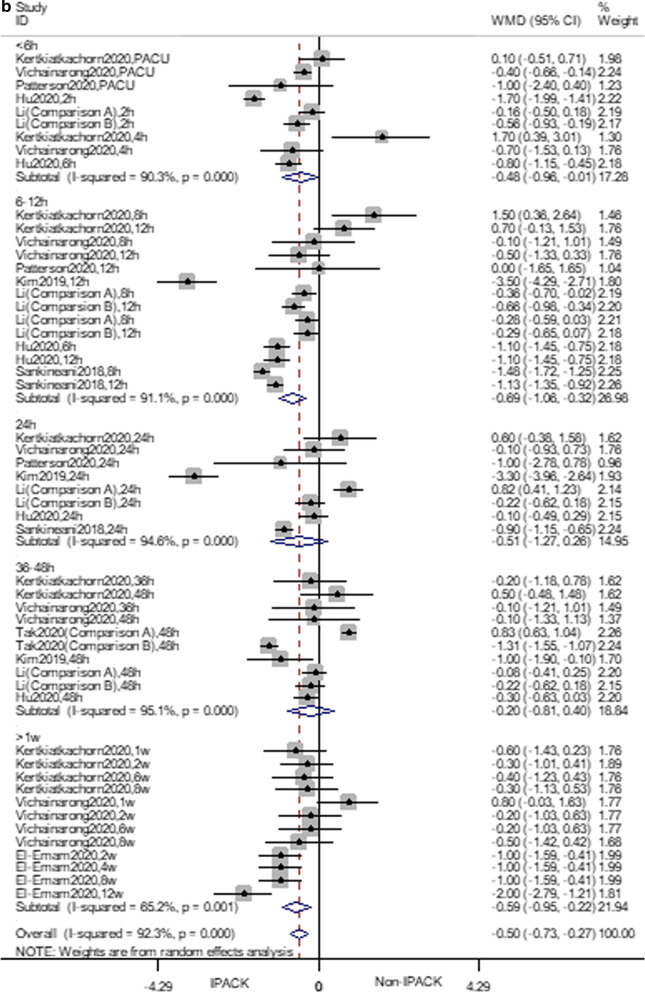

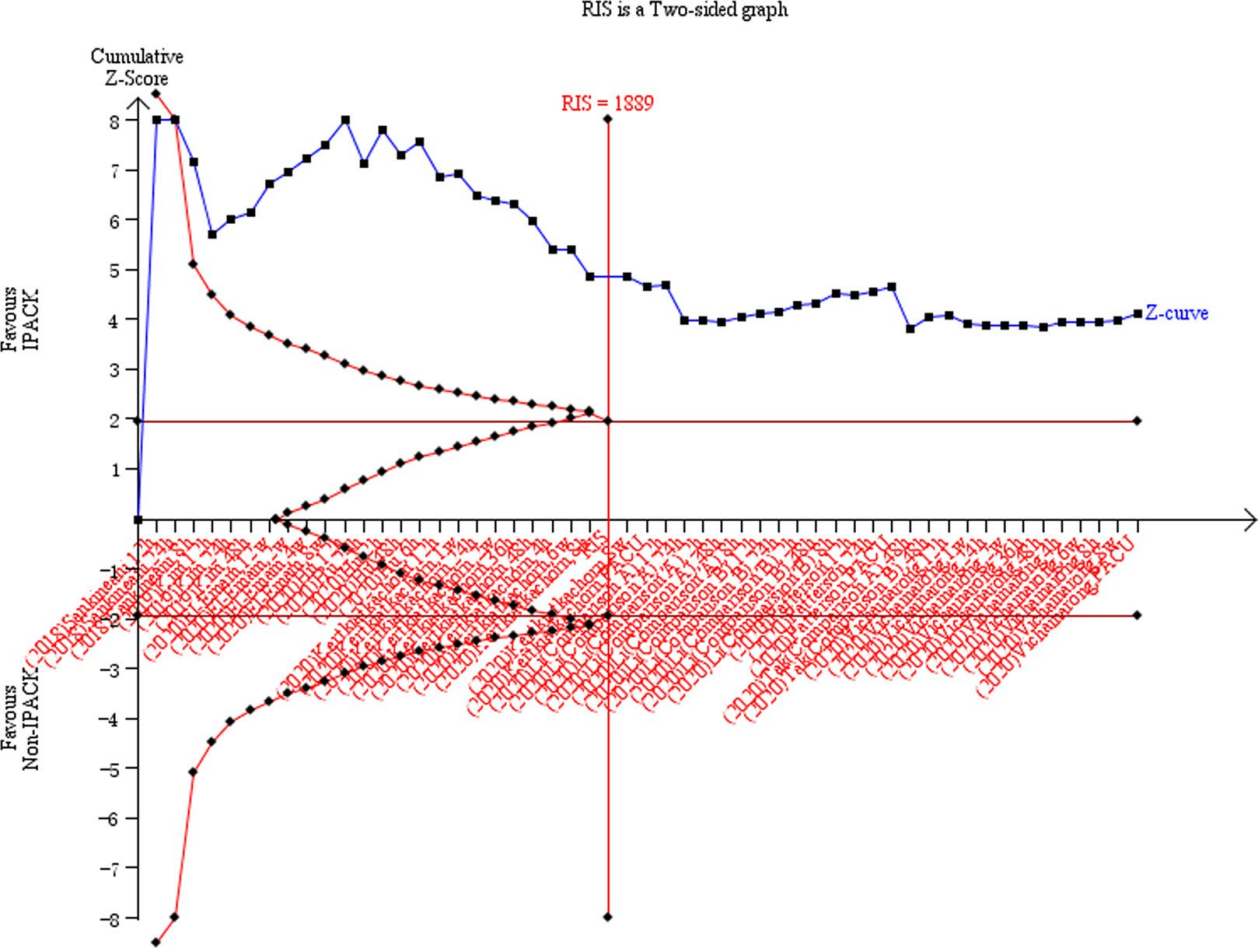
Forest plots **a** forest plot of pain, at ambulation; **b** trial sequential analysis of pain, at ambulation (adjusted boundaries). **c** Trial sequential analysis of pain at ambulation (penalized test)

IPACK reduced ambulation pain scores (WMD = − 0.49 VAS, 95% CI − 0.72 to − 0.26, *p* < 0.0001). Subgroup analysis suggested that IPACK had lower scores within 12 h (2–4 h, WMD = − 0.48, 95% CI − 0.96 to − 0.008, *p* = 0.046; 6–12 h, WMD = − 0.69, 95% CI − 1.06 to − 0.32, *p* < 0.0001), and beyond 1 week (WMD = − 0.59 95% CI − 0.95 to − 0.22, *p* < 0.0001). T.S.A. confirmed the effect of IPACK when performed at a power of 80%. The cumulative z-score crossed the monitoring boundary for the benefit and reached the required sample size (Fig. 3). Due to the inconsistency, the certainty of the evidence was evaluated as moderate (Table 4).

**Table 4:** GRADE, summary of findings, IPACK versus non-IPACK for patients with primary TKA

Patient or Population: Patients with primary total knee arthroplasty Setting: postoperative care in hospital, Egypt (1 trial), India (2 trials), America (3 trials), Thailand (3 trials), China (4 trials) Intervention: The interspace between the popliteal artery and capsule of the knee, IPACK Comparison: Non-IPACK					
Outcome indicator	Importance	Relative effect (95%CI)	No. of Participants (studies)	Quality of the evidence	Comments
Pain at rest (6–12 h)	Critical	− 0.960 (− 1.467, − 0.454)	1309 (13)	⊕⊕⊕○ Moderate ^a^	inconsistency
Pain at ambulation (6–12 h)	Important	− 0.691 (− 1.064, − 0.318)	1223 (13)	⊕⊕⊕○ Moderate ^b^	inconsistency
Morphine consumption (24–48 h)	Critical	− 2.979 (− 5.714, − 0.244)	812 (10)	⊕⊕⊕⊕ High	inconsistency
Morphine requirement (12–24 h)	Important	0.506 (0.309, 0.829)	281 (4)	⊕⊕⊕○ Moderate	inconsistency
Ambulation distances (POD2)	Important	1.743 (0.339, 3.147)	439 (5)	⊕⊕⊕○ Moderate	inconsistency
Sleep disturbance (POD1)	Important	0.388 (0.185, 0.812)	264 (4)	⊕⊕⊕○ Moderate	Small number of participants

GRADE Working Group grades of evidence. High quality: further research is very unlikely to change our confidence in the estimate of effect. Moderate quality: further research is likely to have an important impact on our confidence in the estimate of effect and may change the estimate. Low quality: further research is very likely to have an important impact on our confidence in the estimate of effect and is likely to change the estimate. Very low quality: we are very uncertain about the estimate

^a^Downgraded by two levels due to inconsistency (unexplained high heterogeneity without change results, I^2^ > 75%)

^b^Downgraded by one level due to inconsistency (unexplained high heterogeneity without change results, I^2^ > 50%)

### Pain scores at rest

**Fig. 4:**
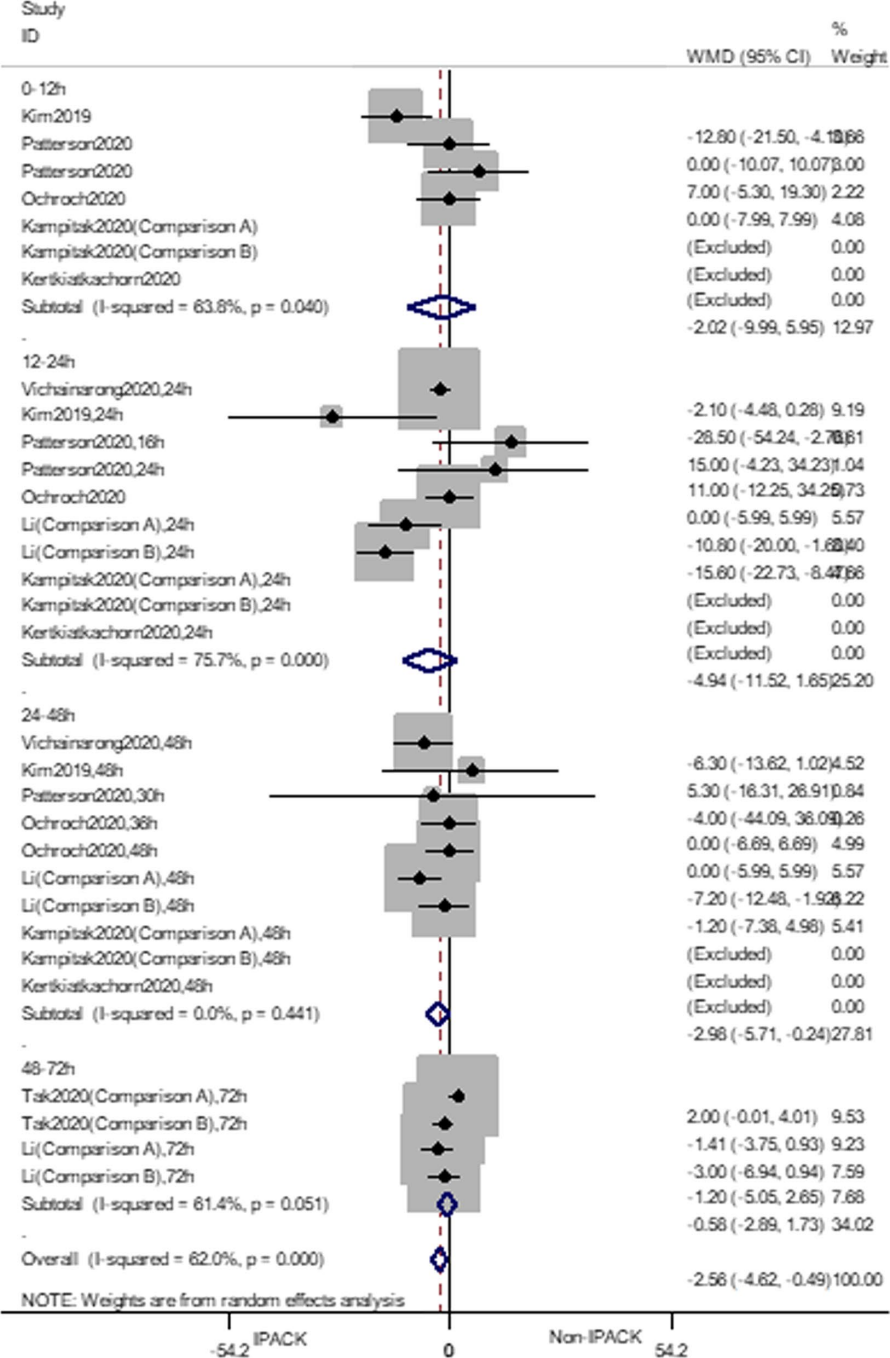
Forest plots **a** forest plot of pain at rest; **b** trial sequential analysis of pain at rest (adjusted boundaries). **c** Trial sequential analysis of pain at rest (Penalized Test)

IPACK was associated with lower pain scores at rest (WMD = − 0.49 VAS, 95% CI − 0.74 to − 0.24, *p* < 0.0001). Subgroup analysis suggested lower rest pain scores with IPACK between 6 and 12 h (WMD = − 0.96, 95% CI − 1.47 to − 0.45, *p* < 0.0001), and beyond 1 week (WMD = − 0.31, 95% CI − 0.62 to − 0.02, *p* = 0.039). T.S.A. confirmed the effect of IPACK, and the cumulative z-score crossed the monitoring boundary for the benefit and reached the required sample size (Fig. 4). Due to the inconsistency, the certainty of the evidence was evaluated as moderate (Table 4).

### Morphine consumption

**Fig. 5:**
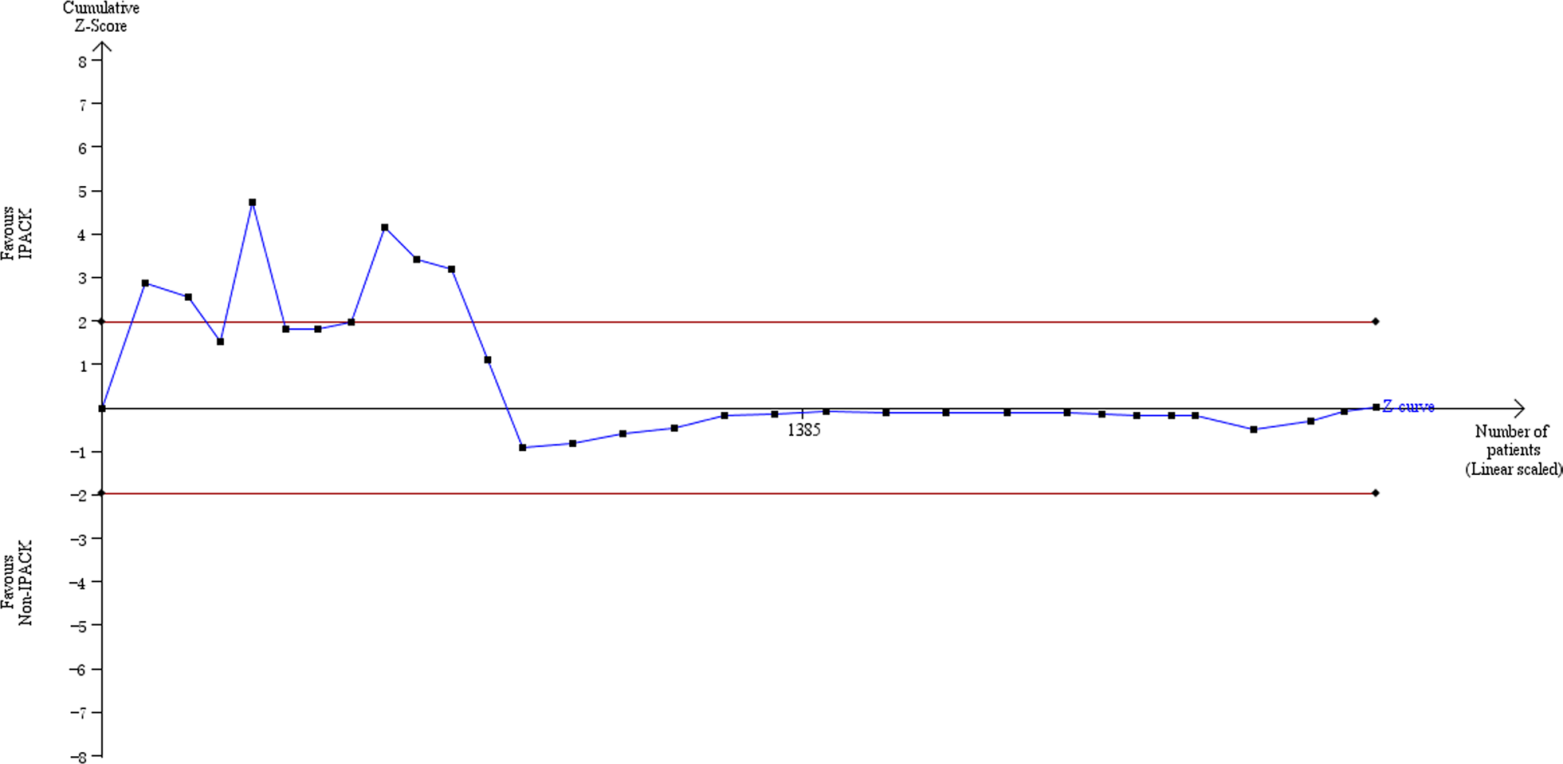
Forest plot of morphine consumption. **a** Forest plot of pain, at rest; **b** trial sequential analysis of morphine consumption (Adjusted Boundaries). **c** Trial sequential analysis of morphine consumption (Penalized Test)

IPACK was associated with a reduction in overall oral morphine consumption (WMD = − 2.56 mg, 95% CI − 4.63 to − 0.49, *p* = 0.015). Subgroup analysis suggested that IPACK reduced the oral morphine consumption from 24 to 48 h postoperatively (WMD = − 2.97 mg, 95% CI − 5.71 to − 0.24, *p* = 0.033). The rate of morphine requirement was reduced with a statistically significant difference in the subgroup of 12 to 24 h (RR = 0.51, 95% CI 0.31 to 0.83, *p* = 0.007). The cumulative z-score failed to cross the benefit’s monitoring boundary or reach the required sample size (Fig. 5). The certainty of the evidence was evaluated as moderate (Table 4).

### Functional recovery

We found that patients who received an additional IPACK could achieve longer ambulation distances during the hospital stay (WMD = 1.12 feet, 95% CI 0.37 to 1.88, *p* = 0.004). A better result was also observed on POD2 (*p* = 0.015). No difference was found on POD0, POD1, or POD3. The synthesized results found that the level of quadriceps muscle strength favored patients in the IPACK group when measured at 0 degrees (WMD = 0.41, 95% CI 0.04 to 0.77, *p* = 0.029). No statistically significant difference was found when patients flexed at 45 or 90 degrees. Moreover, we found no difference regarding the outcomes of ROM (*p* = 0.66) or TUG (*p* = 0.58).

### Complications

Four studies reported the rate of postoperative nausea and vomiting (PONV), and we found no difference in the synthesized rate of PONV between patients who received IPACK and not (*p* = 0.60). The incidence of sleep disturbance was reduced following the use of IPACK (RR = 0.50, 95% CI 0.31 to 0.80, *p* = 0.04). Subgroup analysis found a similar benefit on POD 1 for IPACK using (*p* = 0.012).

### Clinical outcomes

In our study, IPACK was associated with a shorter length of hospital stay while the difference lost significance (*p* = 0.07). No significant difference was found in either operation time (*p* = 0.71) or patient satisfaction (*p* = 0.058).

### Sensitivity analysis

We conducted a sensitivity analysis on all outcomes with moderate-to-high heterogeneity (*I*^*2*^ > 50%) to validate our results. The conclusions remain unchanged in all outcomes, which suggests the stability of our outcomes.

### Publication bias

**Fig. 6:**
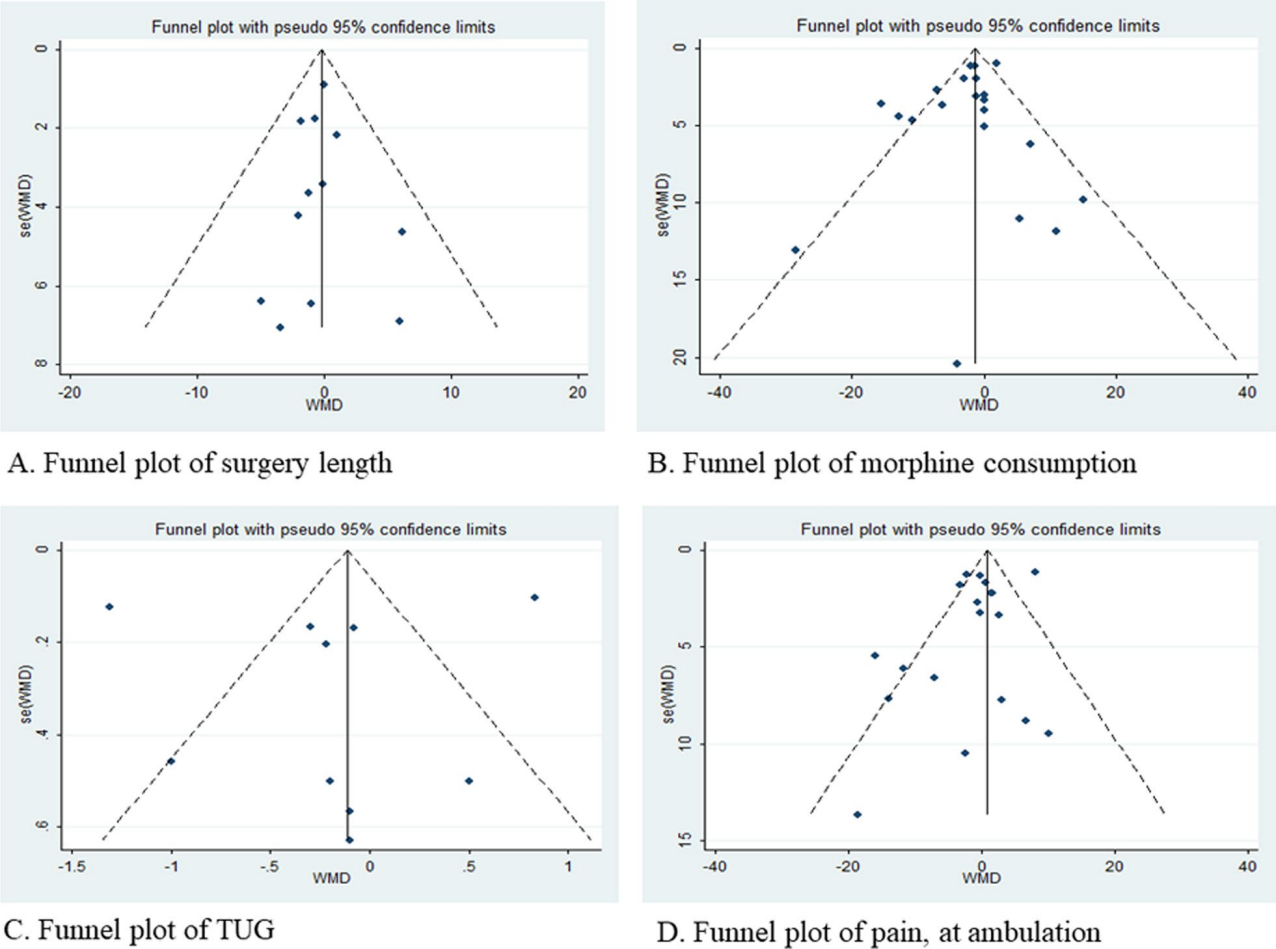
Funnel plots **a** funnel plot of publication bias for the surgery length; **b** funnel plot of publication bias for the morphine consumption; **c** funnel plot of publication bias for the TUG; and **d** funnel plot of publication bias for the pain (at ambulation);

The symmetrical distribution of funnel plots and the *p* value of the egger test both showed no publication bias (Fig. 6). Egger’s test revealed no potential publication bias (*p* > 0.01). No publication bias was found in the trials included.

### Post hoc meta-regression

Meta-regression results found that other nerve blocks can explain 70.08% of heterogeneity, while the others cannot (Additional file 2: Table S1).

## Discussion

Our meta-analysis suggests that the administration of IPACK significantly reduced pain scores when measured at ambulation and rest, and the differences vanished over 24 h. Similarly, IPACK was associated with lower morphine consumption and reduced rate of morphine requirement without increasing the rate of complications. Moreover, functional metrics such as ambulation distances and quadriceps muscle strengthen also favored IPACK, but these differences were marginal and lacked clinical importance.

Due to the rich supply of sensory innervation around the knee joint, patients after TKA always complained about their knee pain. Postoperative pain will increase opioid consumption, prolonged functional immobility, and diminished patient satisfaction. Therefore, adequate analgesia is of paramount importance. Peripheral nerve blocks are effective for TKA pain management. Femoral nerve block targets the anteromedial aspects of the knee, while the weakness of the quadriceps muscle will delay ambulation and increase the risk of fall [4]. The sciatic nerve block provided posterior knee analgesia, while foot drop often occurred [6]. The adductor canal block is gaining popularity by providing better motor preservation and non-inferior analgesia to a femoral nerve block. However, the posterior knee cannot be covered in an isolated adductor canal block [28]. IPACK is a novel but simple procedure that provides adequate analgesia of the posterior capsule of the knee by anesthetizing the articular branches from the sciatic and obturator nerves [29]. Recent evidence confirmed the effect of IPACK in controlling pain, improving physical performance, and decreasing hospital stay [6].

In our analysis, the addition of IPACK improved pain scores at rest and pain scores at ambulation within 24 h, and our results were consistent with previous studies [1, 6, 28]. There was no difference concerning pain VAS scores after 24 h, and possible reasons are that the duration of anesthetic had worn off by one day due to the simple formulation. A new finding was that subgroup analysis suggested the benefits existed beyond one week, suggesting a long-term analgesic effect of IPACK. The associations between immediate postoperative pain and chronic pain after TKA may explain this difference [30]. Of note, the minimal clinically important difference (MCID) for pain scores in TKA was 1.0. The differences brought by the administration of IPACK did not surpass the pre-designated threshold for the clinical importance of 1.0. Possible reasons are that the efficacy of an isolated IPACK was relatively small since the volume was usually 20 to 30 ml and could not infiltrate the membrane. Moreover, there were differences between the architecture of tissue and the properties of injectate and unavoidable variations (i.e., the position of the patient, muscle contraction, needle orientation, etc.) that affect the efficacy of IPACK. Two studies used questionnaires in postoperative pain measurement. Ochroch et al. found reduced average pain scores with IPACK (*p* < 0.01) by the Revised American Pain Society Patient Outcome Questionnaire (APS-POQ-R). Kim et al. [16] found improved analgesia results in the IPACK group (i.e., worst pain scale, least pain scale, severe pain experience on POD1 and POD2) by the patient self-reported questionnaire (Pain OUT). Most studies classified pain as rest and ambulation pain but did not locate the origin of knee pain (i.e., anterior, posterior, medial, lateral). Only two studies reported posterior knee pain [12, 26]. Adequate analgesia following TKA can reduce pain scores and opioid use to prevent complications and facilitate functional recovery. Our study also found positive results regarding reduced morphine consumption. Our results were consistent with previous studies [31–33]. However, the differences failed to reach MCID since a reduction of 40% in opioid usage were considered clinically relevant differences after TKA.

As for functional recovery, patients receiving an additional IPACK block performed better than those who did not receive regarding ambulation distances and muscle strength, indicating that the IPACK might provide potential additional functional improvement when combined with other regional anesthesia methods but was not associated with any meaningful clinical benefits. Possible reasons are that the improved pain experience can promote early ambulation, and decreased opioid consumption reduces adverse events, thereby improving patients’ functional outcomes. Moreover, several studies used questionnaires in measuring knee recovery. Li et al. [3] reported the Knee Society Score (KSS) at discharge, and in three months, they found similar results with IPACK and without. El-Emam et al. [13] found superior Western Ontario and McMaster Universities Osteoarthritis Index (WOMAC) scores in the IPACK group (2–12 weeks), while Li [3] found no difference (at discharge, three months). In general, a marginally better benefit on functional ability was found in our study, which required more data for clarification.

Complications were rare when applying IPACK into the multimodal analgesia pain management, which also proved the safety of IPACK in our study. Possible reasons are that effective pain control reduced opioid consumption and minimized associated side effects further. Some complications cannot be quantitatively synthesized. Li et al. reported two patients with slight numbness on the operative lower extremity with IPACK [3]. Tak et al. found two cases of cardiac events with IPACK, which they believed was not ascribed to IPACK [10]. Kertkiatkachorn et al. used the VAS to assess the severity of PONV and dizziness and found no difference [7]. Moreover, improved sleep quality was found in the IPACK group on POD1 in our study, which improved knee pain and mitigated anxiety [34]. Studies demonstrated that patient satisfaction is not a sole reliable proxy for pain relief and functional recovery outcomes since the factors affecting satisfaction are complex [35, 36]. However, overall patient satisfaction was similar in our study.

New techniques of IPACK have been discussed in several studies. Kampitak et al. [26] compared the effect of proximal IPACK with distal IPACK and found a lower rate of posterior knee pain in the proximal IPACK group. Possible explanations were that the injection point of the proximal IPACK block was closer to the popliteal plexus and promoted the spread of local anesthetic [38–40].

This study has some limitations. First, there was relatively high heterogeneity in several outcomes. However, sensitivity analysis was carried out, and all outcomes’ conclusions remained unchanged. Second, the control groups were not a placebo, and these interventions were various. A network meta-analysis would be of extreme interest. In addition, considering the small sample size and low incidence of the complications, we also designed similar RCTs with a larger sample size to evaluate complications of IPACK (ChiCTR2000032963, ChiCTR2000032964, ChiCTR2000032965, ChiCTR2000032966).

## Conclusions

Our trial demonstrated significantly better pain scores, opioid consumption, and functional outcomes after using IPACK. However, the differences were small and lacked clinical importance, suggesting that IPACK was a relatively effective perioperative analgesia method. Taken as a whole, the current results support the performance of IPACK as a supplement analgesic method. Further investigation with larger samples would lend further insight and implications on the use of IPACK.

## Supplementary Information

**Additional file 1**. The search strategy of our study.

**Additional file 2**. The results of meta-regression.

## Data Availability

The data could be retrieved from the corresponding authors if necessary.

## Abbreviations

TKA: Total knee arthroplasty
ACB: Adductor canal block
IPACK: Interspace between the popliteal artery and posterior capsule of the knee
PAI: Periarticular injection
LIA: Local infiltration analgesia
VAS: Visual analog scale
95% CI: 95% Confidence intervals
FNB: Femoral nerve block
POD: Postoperative day
PRISMA: Preferred Reporting Items for Systematic Reviews and Meta-Analyses
RCT: Randomized controlled trial
R.O.M.: Range of motion
RR: Relative risks
WMD: Weight mean difference
NRS: Numeric rating scale
