# Correction: Analgesic efficacy of adding the IPACK block to multimodal analgesia protocol for primary total knee arthroplasty: a meta-analysis of randomized controlled trials

**DOI:** 10.1186/s13018-022-03460-3

**Published:** 2022-12-26

**Authors:** Tang xiumei, Lai Yahao, Du Siwei, Ning Ning

**Affiliations:** 1 West China School of Nursing, Sichuan University, #37 Guoxue Road, Chengdu, 610041 People’s Republic of China; 2 Department of Orthopedics, West China Hospital, Sichuan University, #37 Guoxue Road, Chengdu, 610041 People’s Republic of China

**Correction to:** **Journal of Orthopaedic Surgery and Research (2022) 17:249** 10.1186/s13018-022-03266-3

Following the publication of the original article [1], the authors reported that the affiliations of all authors are incorrectly published.

The correct affiliations are shown below.

The original article has been corrected.
