# Supplementary material for: Analgesic efficacy of adding the IPACK block to multimodal analgesia protocol for primary total knee arthroplasty: a meta-analysis of randomized controlled trials
Source: J Orthop Surg Res. 2022 Sep 29;17:429. doi: 10.1186/s13018-022-03266-3 (PMC9523917; doi:10.1186/s13018-022-03266-3)
Supplement: Supplementary file 1 — Additional file 1. The search strategy of our study. [file 13018_2022_3266_MOESM1_ESM.docx]

**Search Strategy**

(((((((((((((((((((((((((((((((((((Arthroplasty, Replacement, Knee[MeSH Terms]) OR Knee Prosthesis[MeSH Terms]) OR knee replacement arthroplasty[Text Word]) OR total knee arthroplasty[Text Word]) OR total knee[Text Word]) OR TKA[Text Word]) OR TKR[Text Word]) OR total knee replacemen[Text Word]) OR knee prosthesis[Text Word]) OR knee implantation[Text Word]) OR knee implant[Text Word]) OR knee implants[Text Word]) OR knee prosthesis[Text Word]) OR knee joint replacement[Text Word]) OR knee joint arthroplasty[Text Word]) OR Knee Replacement Arthroplasties[Text Word]) OR Total Knee Replacements[Text Word]) OR Knee Prostheses[Text Word]) OR Knee endoprosthesis[Text Word]) OR Knee endoprostheses[Text Word]) OR Knee joint arthroplasty[Text Word]) OR Knee joint arthroplasties[Text Word]) OR knee joint prosthesis[Text Word]) OR knee joint prostheses[Text Word]) OR knee prosthetic[Text Word]) OR Knee endoprosthetic[Text Word]) OR knee joint prosthetic[Text Word]) OR Knee joint endoprosthetic[Text Word]) OR knee prosthetics[Text Word]) OR Knee endoprosthetics[Text Word]) OR knee joint prosthetics[Text Word]) OR Knee joint endoprosthetics[Text Word]) OR Knee replacement[Text Word]) OR Knee replacements[Text Word]) OR knee arthroplasty[Text Word]) OR knee arthroplasties[Text Word]

and ((((adductor canal block[Text Word] OR saphenous nerve block[Text Word] OR peripheral nerve block[Text Word] OR ACB[Text Word])

and (IPACK[Text Word] OR interspace between the popliteal artery and the posterior capsule of the knee[Text Word])

and

((((((((((((((((((((randomized controlled tria[Publication Type]) OR randomized controlled trials as topic[MeSH Terms]) OR random allocation[MeSH Terms]) OR double-blind method[MeSH Terms]) OR single-blind method[MeSH Terms]) OR placebos[MeSH Terms]) OR random*[Text Word]) OR ramdom*[Text Word]) OR ramdon*[Text Word]) OR randon*[Text Word]) OR rct[Text Word]) OR rct’s[Text Word]) OR rcts[Text Word]) OR RCT[Text Word]) OR placebo*[Text Word]) OR random*[Text Word]) OR compare*[Title]) OR versus[Title]) OR vs[Title])) OR ((((mask*[Text Word]) OR blind*[Text Word])) AND ((((single[Text Word]) OR double[Text Word]) OR treble[Text Word]) OR triple[Text Word]))

**Forest Plots**


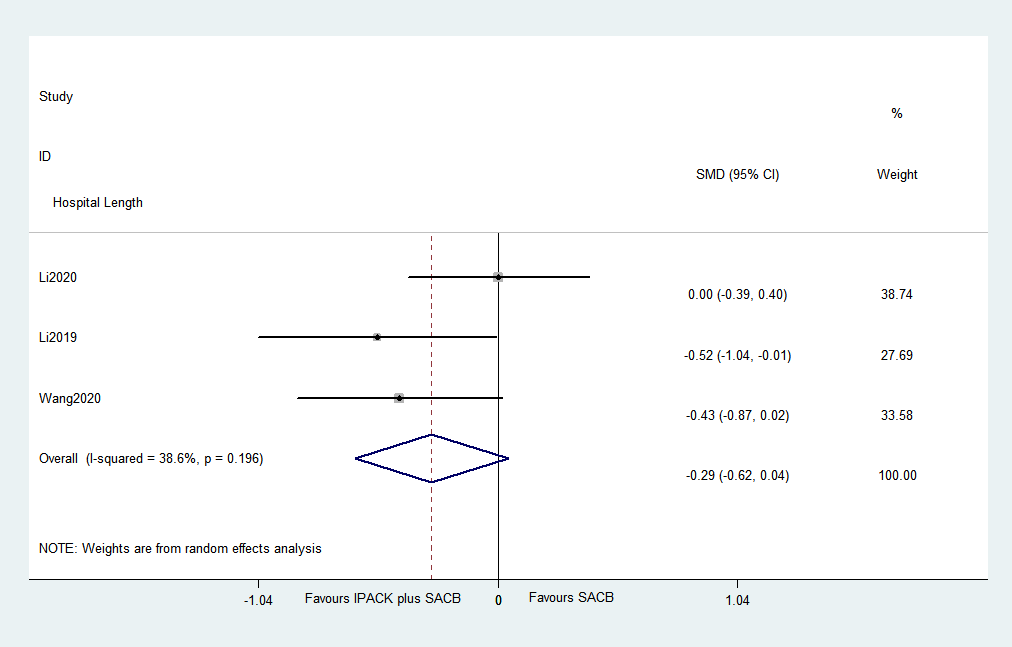


Hospital Length


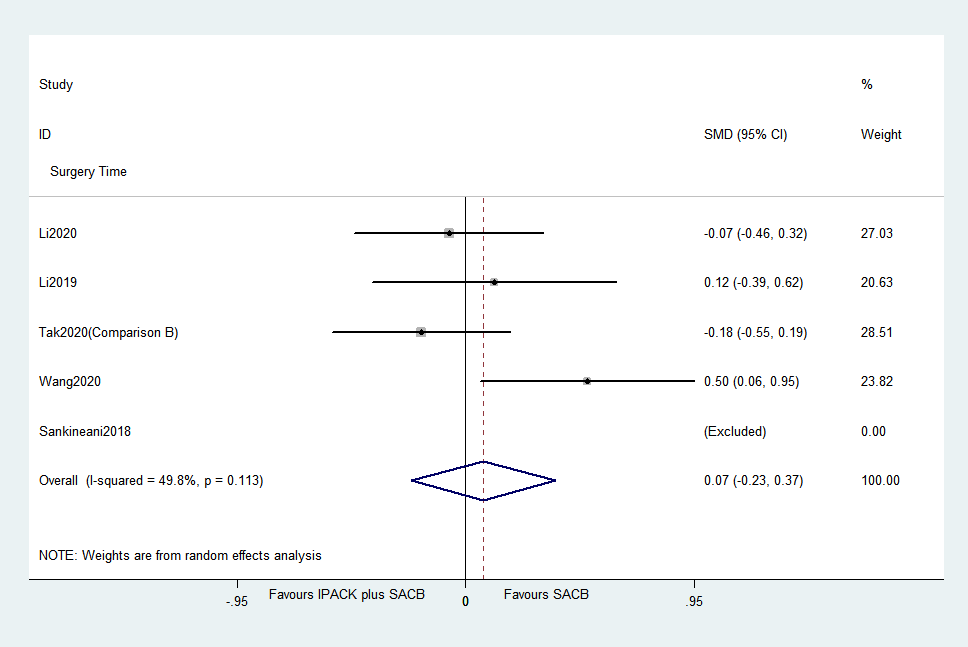


Length of Surgery


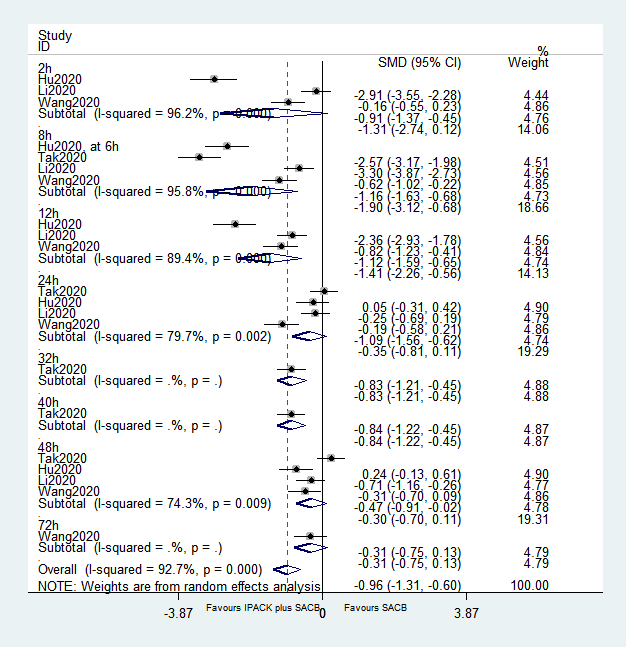


Visual Analogue Scale Scores, at Rest


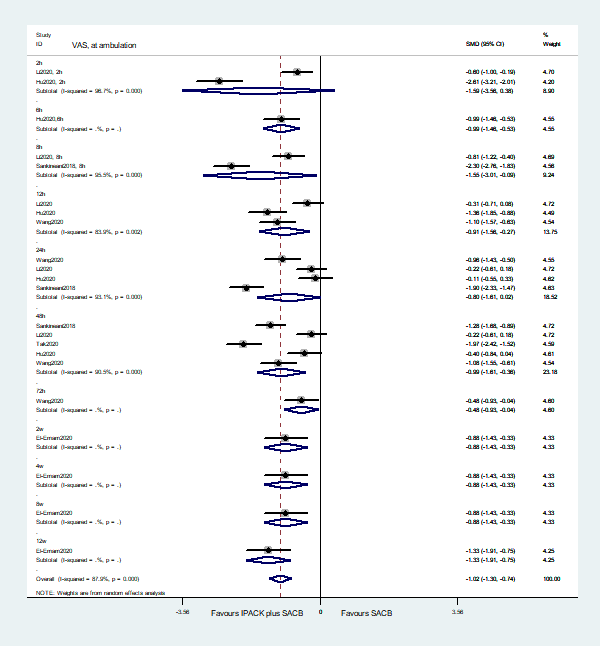


Visual Analogue Scale Scores, at Ambulation


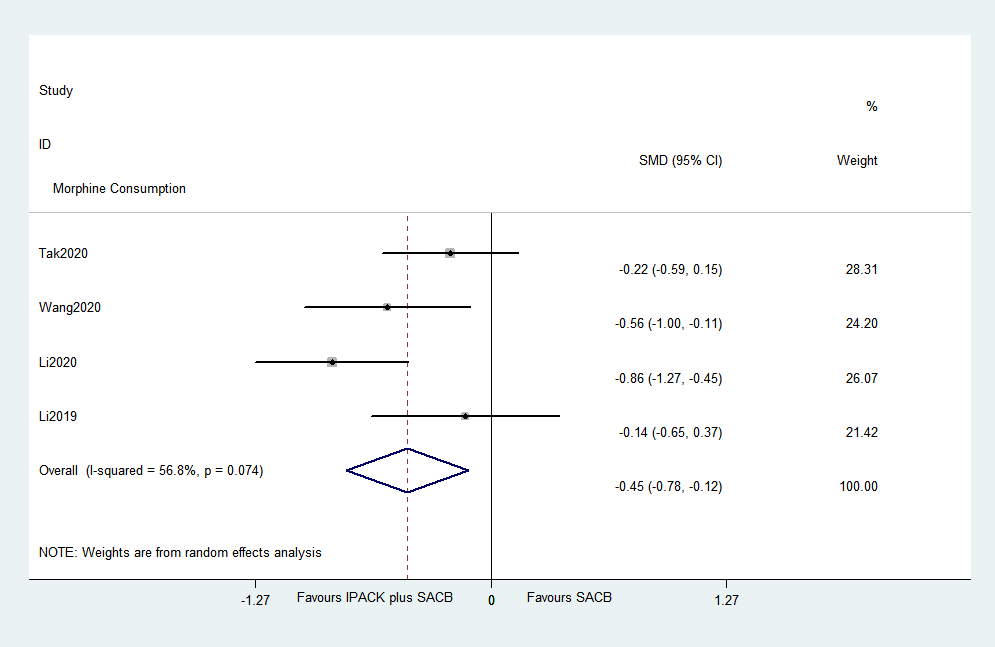


Morphine Consumption


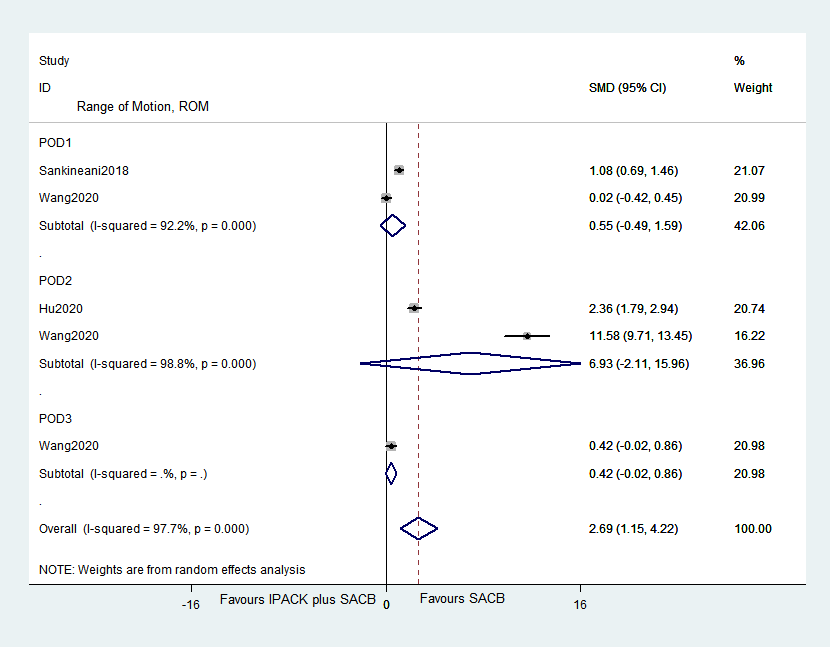


Range of Motion


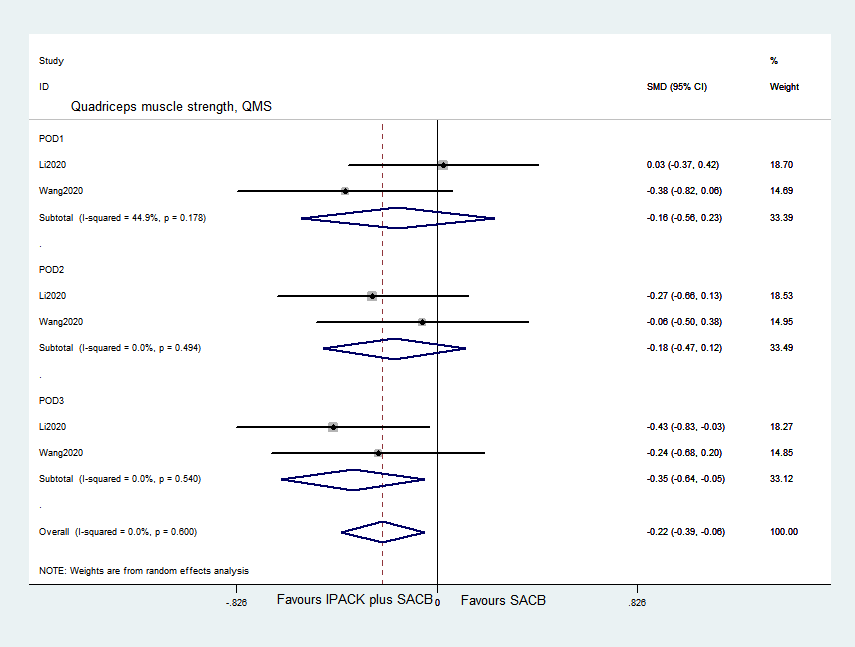


Quadricep Muscle Strength


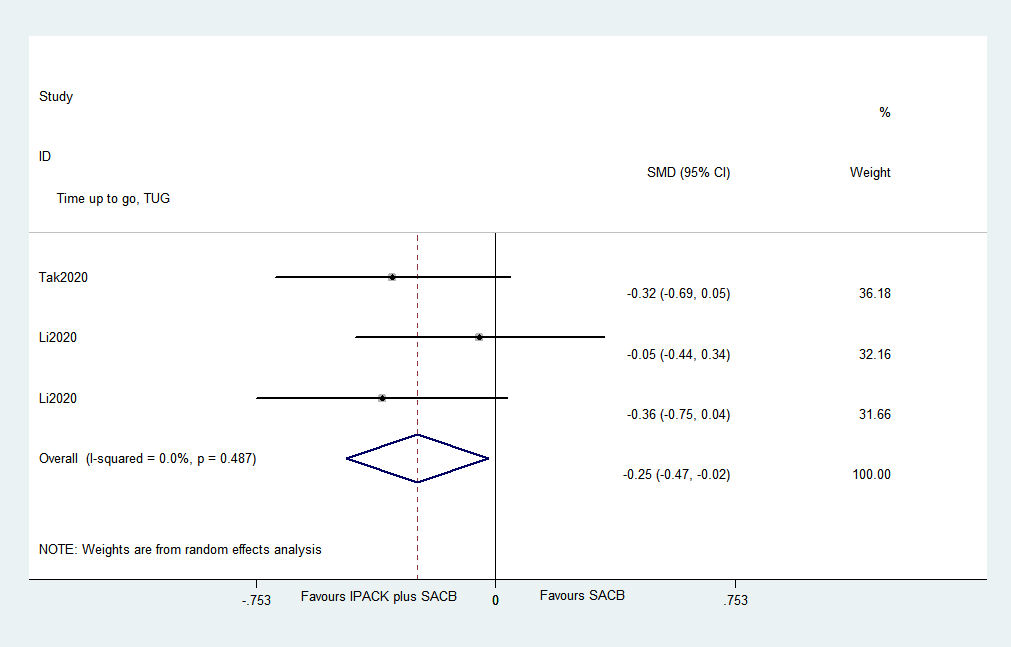


Time up to Go Test


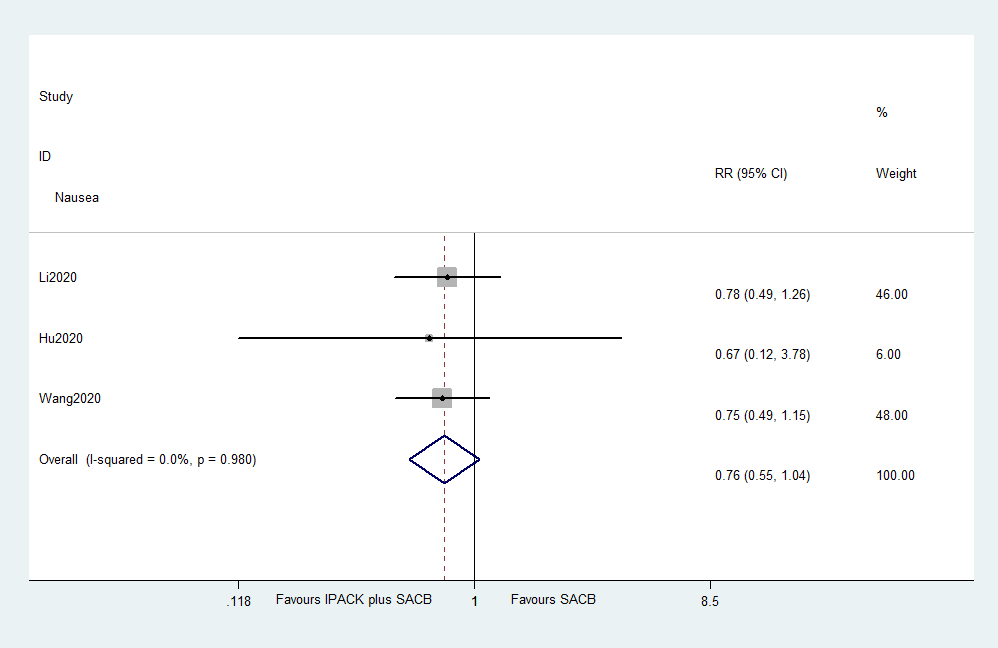


Nausea

**Sensitivity Analysis**

Visual Analogue Scale Scores, at Rest


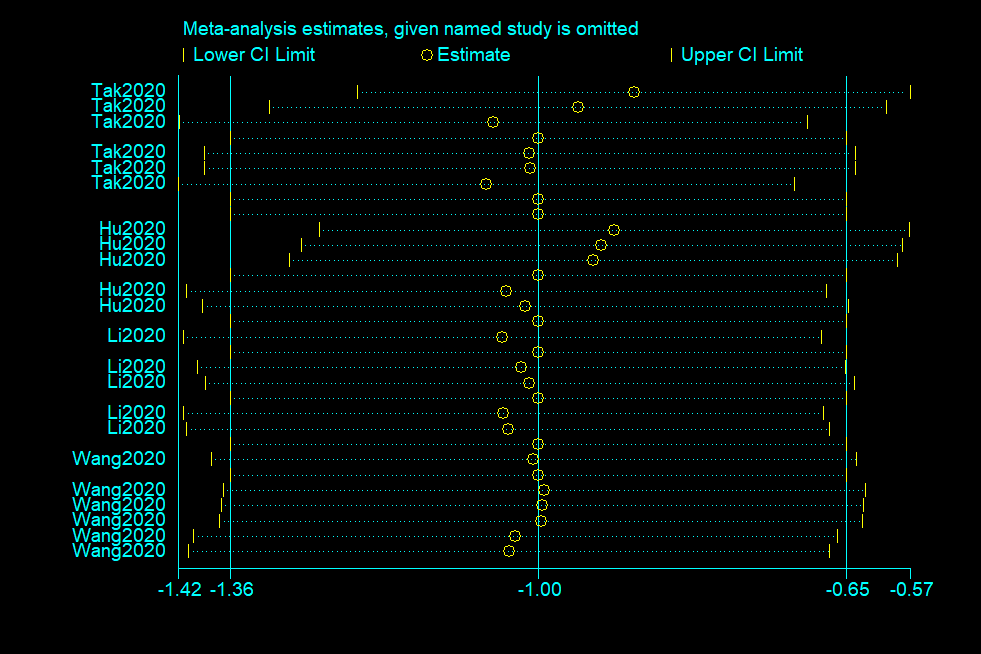


Visual Analogue Scale Scores, at Ambulation


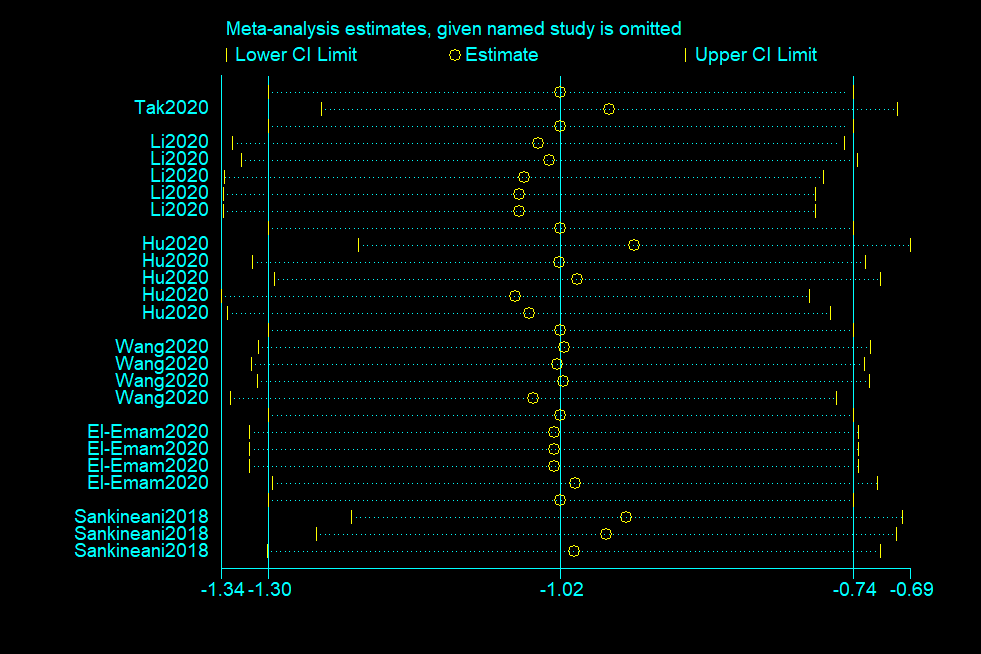


A1. Visual Analogue Scale Scores, at Rest: Funnel Plots


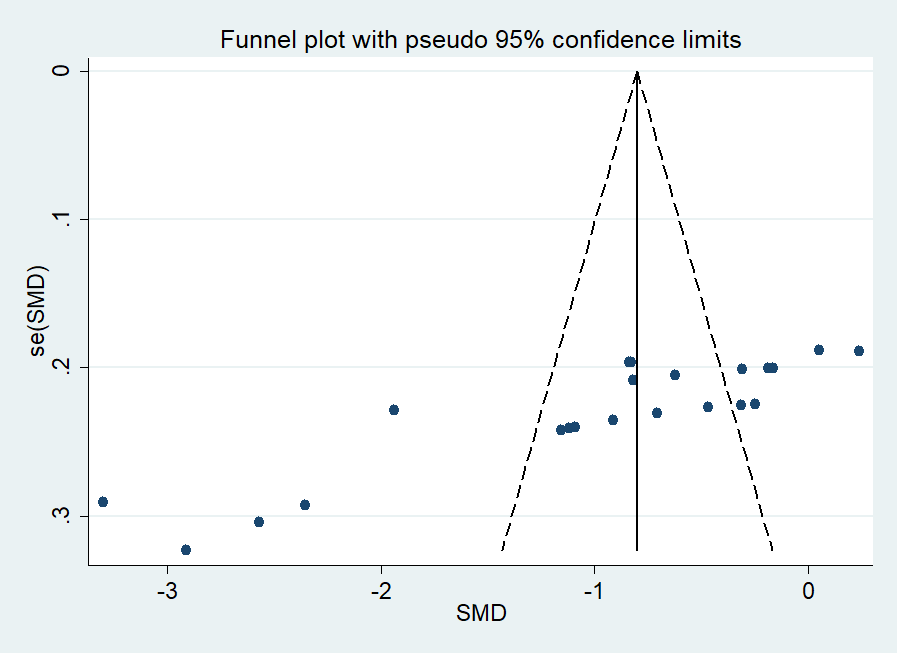


A2. Visual Analogue Scale Scores, at Rest: Egger Test


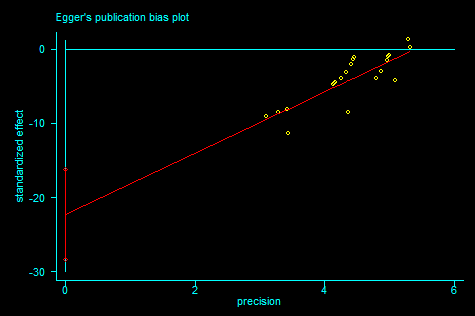

A3. Visual Analogue Scale Scores, at Rest: Trim and Fill Method

B1. Visual Analogue Scale Scores, at Ambulation: Funnel Plots


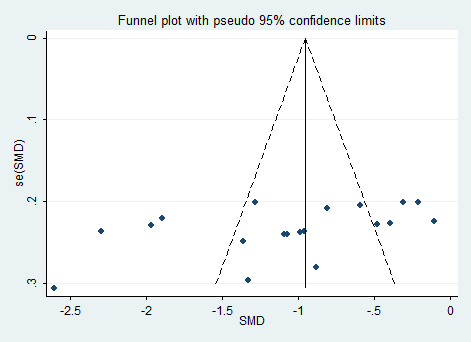


B2. Visual Analogue Scale Scores, at Ambulation: Egger Test


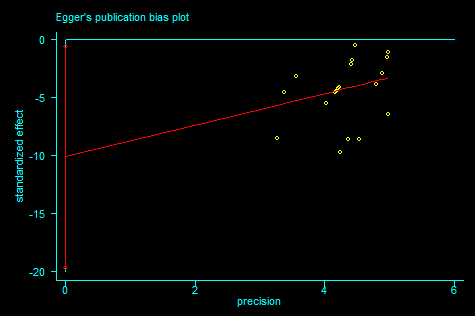

B3. Visual Analogue Scale Scores, at Ambulation: Trim and Fill Method
