# Supplementary material for: Analgesic efficacy of adding the IPACK block to multimodal analgesia protocol for primary total knee arthroplasty: a meta-analysis of randomized controlled trials
Source: J Orthop Surg Res. 2022 Sep 29;17:429. doi: 10.1186/s13018-022-03266-3 (PMC9523917; doi:10.1186/s13018-022-03266-3)
Supplement: Supplementary file 2 — Additional file 2. The results of meta-regression. [file 13018_2022_3266_MOESM2_ESM.docx]

Supplement file. The results of meta-regression (interaction)

The results of meta-regression (CACB)
